# Recent Advances in the Application of Cucurbitacins as Anticancer Agents

**DOI:** 10.3390/metabo13101081

**Published:** 2023-10-14

**Authors:** Bartłomiej Zieniuk, Magdalena Pawełkowicz

**Affiliations:** 1 Department of Chemistry, Institute of Food Sciences, Warsaw University of Life Sciences, 02-776 Warsaw, Poland; bartlomiej_zieniuk@sggw.edu.pl; 2 Department of Plant Genetics, Breeding and Biotechnology, Institute of Biology, Warsaw University of Life Sciences, 02-776 Warsaw, Poland

**Keywords:** cucurbitacin, anticancer mechanisms, molecular targets, mechanism of action, biological activity

## Abstract

Cucurbitacins are tetracyclic triterpenoid secondary metabolites, widely distributed in the *Cucurbitaceae* family. These bitter-tasting compounds act primarily as defense mechanisms against external injuries, and thus against herbivores, and furthermore, they have also found use in folk medicine in the treatment of various diseases. Many studies have acknowledged significant biological activities of cucurbitacins, such as antioxidant and anti-inflammatory activities, antimicrobial properties, or antitumor potential. Overall, cucurbitacins have the ability to inhibit cell proliferation and induce apoptosis in various cancer cell lines. Both in vitro and in vivo studies were performed to evaluate the anticancer activity of varied cucurbitacins. Cucurbitacins offer a promising avenue for future cancer treatment strategies, and their diverse mechanisms of action make them attractive candidates for further investigation. The aim of the present study is to shed light on the chemical diversity of this group of compounds by providing the sources of origin of selected compounds and their chemical structure, as well as insight into their anticancer potential. In addition, within this paper molecular targets for cucurbitacins and signalling pathways important for cancer cell proliferation and/or survival that are affected by the described class of compounds have been presented.

## 1. Introduction

The vast pharmacological repertoire of natural products has been explored in the relentless search for innovative approaches to cancer. Within this field, cucurbitacins, a group of structurally diverse triterpenoids, have emerged as intriguing molecules with promising anticancer potential. These compounds, which are abundant in plants of the *Cucurbitaceae* family and other botanical sources, have attracted considerable attention due to their diverse activities, which include anti-inflammatory, antiviral, and, in particular anticancer effects [1]. The basic functions of cucurbitacins are defense against herbivores due to their bitter taste, and insect repellent activity [2]. The use of cucurbitacins in traditional medical systems across cultures underline their historical relevance as therapeutic agents. However, it is modern scientific advances that have elucidated the intricate mechanisms underlying their pharmacological actions. Cucurbitacins exhibit a striking ability to modulate key signalling pathways within cancer cells, affecting processes such as cell cycle regulation, apoptosis, and angiogenesis [1,3]. Numerous studies have demonstrated the anticancer potential of cucurbitacins. The fight against cancer continues to require the exploration of novel therapeutic avenues. Cancer is the second leading cause of death in the United States and a worldwide public health problem [4]. During the 2014–2018 years, the following overall cancer incidence rates were observed: 497 per 100,000 and 431 per 100,000 for men and women, respectively [5]. In addition, there were approximately 19.3 million new cancer cases and almost 10.0 million cancer deaths in 2020 [6]. Cucurbitacins, a group of highly oxidized tetracyclic triterpenoids abundant in various plant species, have emerged as promising candidates for anticancer interventions [1,3]. Despite their immense therapeutic potential, cucurbitacins face challenges related to their natural abundance, limited availability, and hydrophobic nature [1,3]. As a result, researchers have turned to chemical synthesis to overcome these limitations and enhance their potential in clinical settings. In addition, innovative drug delivery strategies, such as nanocarrier systems, have emerged to improve the solubility, stability, and targeted delivery of cucurbitacin to tumor sites [3,7]. Further elucidation of the intricate molecular mechanisms and exploration of synergistic effects with other small-molecule drugs may expand the therapeutic applications of cucurbitacins [3,8].

To date, more than 100 species of cucurbitacins and their derivatives have been isolated from about 30 genera of the *Cucurbitaceae* family (for example, cucumber, pumpkin, melon, watermelon, and momordica) [9]. In addition, there are several cucurbitacins discovered in other families, for instance, *Thymelaeaceae* [10], *Brassicaceae*, *Datiscaceae*, *Primulaceae*, and *Rubiaceae* [11]. Still, these chemicals are not only limited to plants but are also present in fungi of the *Tricholomataceae* family [12,13].

**Figure 1:**
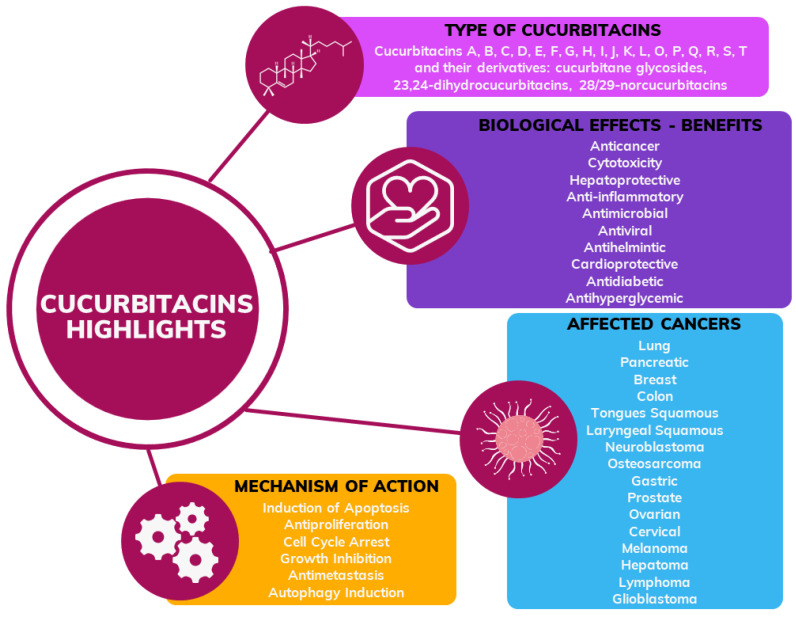
General scheme of information about cucurbitacins including type, biological effects, type of cancer and mechanism of action confirmed in the previous research.

This comprehensive review aims to provide an overview of the key aspects of the potential of cucurbitacins (Figure 1) as anticancer agents, covering their molecular mechanisms, pharmacological actions and challenges in their use. By synthesizing information from preclinical studies, mechanistic studies and clinical trials, we aim to provide a solid resource for researchers, clinicians and pharmaceutical developers. Our goal is to establish a solid foundation for the strategic exploration of cucurbitacins as potential adjuncts to conventional anticancer therapies and the design of innovative anticancer strategies. In this way, we aim to contribute to the development of targeted therapeutic interventions based on cucurbitacin science.

## 2. Chemical Diversity of Cucurbitacins and Their Occurrence in Various Organisms

**Figure 2:**
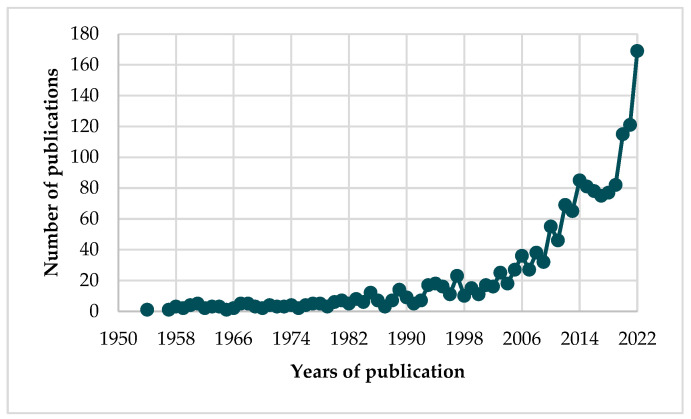
Number of publications on cucurbitacins (Source: Scopus; *n* = 1646).

Taking into account the Scopus database, the first article on cucurbitacins was published in 1954 [14]. Analyzing the period 1954–2022, 1646 articles were published (Figure 2), of which 1316 were original research articles and 248 were review articles. Figure 2 shows the number of publications on cucurbitacins. Since the 2000s an increase in the number of publications on this topic has been observed, while in 2014–2019 the number of articles remained at around 80 articles. In recent years, a dynamic increase in interest in cucurbitacins has been observed, with the following number of publications: 115 in 2020, 121 in 2021, and 169 in 2022.

**Figure 3:**
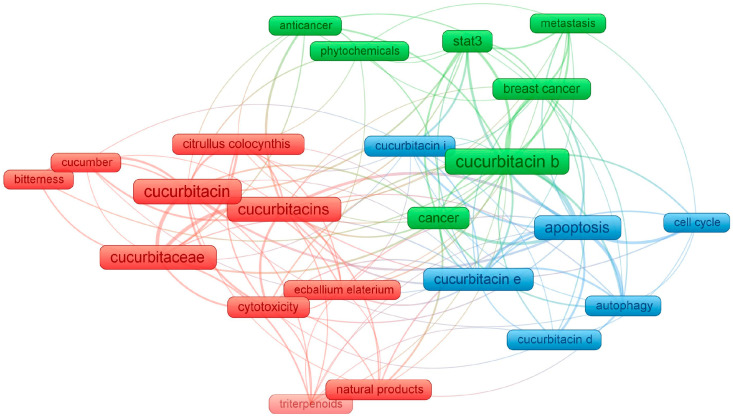
Network visualization of the keywords based on their co-occurrence, obtained by VOSviewer, where the size of each frame is proportional to the number of occurrences.

By setting a minimum number of occurrences of a keyword at 15, the network of 23 keywords is presented in Figure 3. Firstly, it can be concluded that cucurbitacin B is the most frequently researched substance among the entire group of these compounds. Among other tested compounds, research is often undertaken on cucurbitacins E, D, and I. Moreover, three clusters are distinguished, i.e., the first with red labels referred to the isolation of these natural compounds from the *Cucurbitaceae* family, especially from *Citrullus colocynthis* and *Ecballium elaterium*. The authors in the used keyword sets also mentioned the bitterness and potential cytotoxicity of cucurbitacins. Both green and blue clusters referred to the anticancer potential of cucurbitacins, and thus show the interest of the scientific community in these issues. This visualization is primarily seen as a combination of the subject of these chemical compounds and medical terminology closely related to cancer research (“metastasis”, “autophagy”, “cell cycle” or “apoptosis”). The presented relationships, therefore the use of cucurbitacins in cancer research will be described in more detail in the subsequent chapters of this review article.

**Figure 4:**
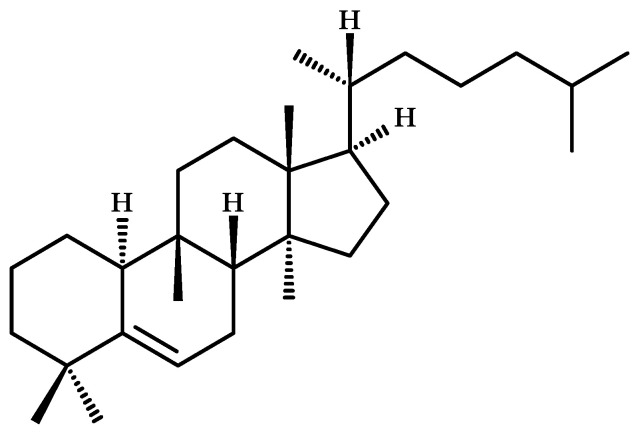
Chemical structure of cucurbita-5-ene (19(10→9β)-abeo-10α-lanost-5-ene), precursor of cucurbitacins.

Most cucurbitacins are chemically classified as tetracyclic triterpenoids and are derived from the unsaturated hydrocarbon cucurbita-5-ene (19(10→9β)-abeo-10α-lanost-5-ene) (Figure 4) [15], which is composed of six isoprene units with the molecular formula C_30_H_51_ [16].

**Figure 5:**
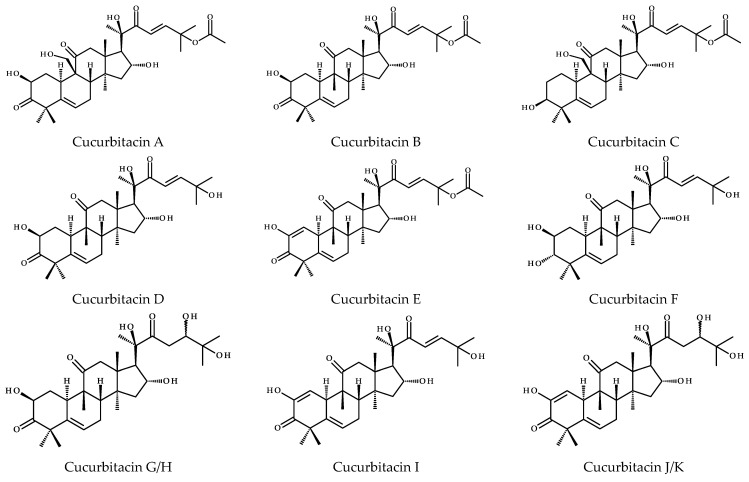

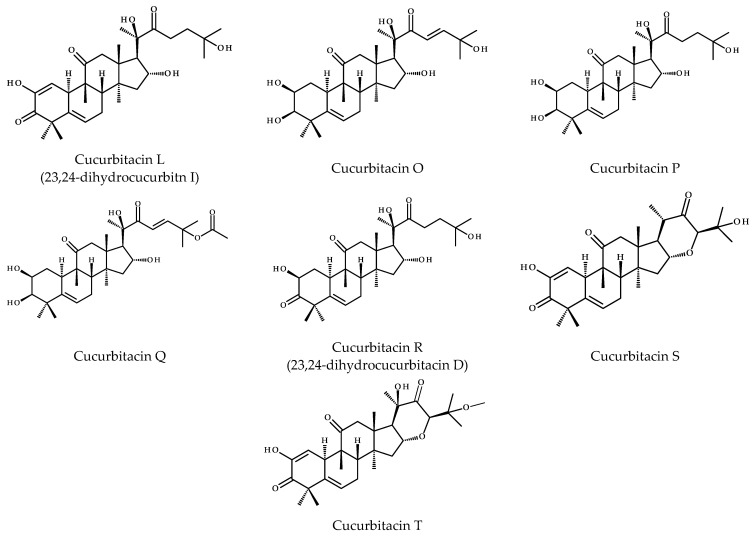
Chemical structures of cucurbitacins A–T.

To date, more than eighteen different basic cucurbitacins have been discovered and described; and the variants are named with the capital letters A-T (Figure 5). Some of them occur as glycosides and contain several hydroxyl, carbonyl, and acetoxyl functional groups [11]. Sixteen chemical structures representing cucurbitacins A-T in the aglycone form are presented below in Figure 5.

Cucurbitacins are highly oxygenated chemical compounds, and their structures typically differ in terms of the number of hydroxyl groups, for instance, cucurbitacin A vs. B, that is 9-hydroxymethyl vs. 9-CH_3_ substitutions or in the case of cucurbitacins B and Q, where the latter has the C3-hydroxyl group. Cucurbitacin D is considered to be the most abundant member of this group [16], which similar to cucurbitacins F, I, and O lacks the acetyl group at the 25-OH. The diversity of these compounds also results from the different degrees of unsaturation. Common to all compounds is the unsaturated bond at C5 derived from the cucurbita-5-ene structure, but in addition, other compounds have extra bonds between C1 and C2 (e.g., cucurbitacins I, J, or S), as well as between C23-C24, i.e., cucurbitacin I and its saturated derivative—23,24-dihydrocucurbitn I (referred to as cucurbitacin L) or cucurbitacin D and 23,24-dihydrocucurbitacin D (cucurbitacin R). Interestingly, cucurbitacins G and H have the same structure, except for the difference in stereochemistry at C24, and the same is observed for cucurbitacins J and K. Furthermore, cucurbitacins S and T have an extra ring due to the cyclization between C16 and C24. The former also occurs in equilibrium with its hemiacetal [16].

There are many other cucurbitacins that differ in their structure from the chemical compounds described above. A ring A modified by the loss of the methyl groups (C28 or C29), which are attached to C4, becomes aromatic and these compounds are called 28/29-norcucurbitacins [17]. Going further, neocucurbitacins A and B isolated from *Luffa operculata* are examples of lactone-type cucurbitacins [18]. The structures of cucurbitacins and their derivatives are generally isolated and described as glycosides, in detail they are mainly derived from glucose (glucopyranose), where the *O*-glycosidic bond comes from the C2-hydroxyl group of the cucurbitacin, or less often from the C3-hydroxyl group [16].

The *Cucurbitaceae* family includes 130 genera and 800 species, of which about 150 of them are widely cultivated. Approximately 30 species play a major role in food production and countries such as China, Turkey, and the USA are the leading producers of cucurbits [15]. *Cucurbita pepo* and *C. maxima* Duchesne are probably the most commonly used cucurbits in folk medicine in different regions of the world. Over the years, both seeds, leaves, fruits, and flowers were applied in the treatment of digestive problems, anemia, high blood pressure, obesity, and skin diseases [19]. The phytochemicals found in *Cucurbita* plants are mainly carotenoids and vitamin E, but other authors also describe small amounts of flavonoids and phenolic acids [19]. As the name suggests, plants from the *Cucurbitaceae* family are the source of cucurbitacins, and examples of plants from this family containing these chemical compounds are presented in Table 1.

Cucurbitacins and their derivatives were isolated from a wide variety of cucurbits around the world, such as the roots of the Namib Desert endemic melon (*Acanthosicyos horridus*) [20], the leaves and branches of the Javan cucumber (*Alsomitra macrocarpa*) from the tropical forests of the Indonesian islands and the Malay Archipelago [21], and the bulb of *Bolbostemma paniculatum* from southern China [22]. From the roots of *Bryonia cretica*, used in Egyptian natural medicine, the authors were able to isolate a wide range of compounds, i.e., cucurbitacins B, D, E, G, H, and J, as well as, novel chemicals, such as bryoniaosides A and B, cucurbitane-type triterpene diglycosides linked to L-rhamnose and D-glucose [23,24]. The fruits and roots of *Cayaponia racemosa* and *C. tayuya* were the source of cucurbitacins B, D, and P or their saturated derivatives (cucurbitacin R, 23,24-dihydrocucurbitacins B and F) or deacetylpicracin [25,26,27].

Bitter apple or bitter cucumber are the name given to *Citrullus colocynthis*, a plant that has been widely used in folk medicine for centuries, and is now a very popular subject of research to evaluate the biological properties and toxicity of its cucurbitacins. Organic solvent-based extraction from both leaves and fruits of *C. colocynthis* was an effective method to isolate the described compounds [28,29]. The leaves were rich in cucurbitacin B and cucurbitacin E 2-*O*-β-D-glucopyranosides [29]. The fruits found their application in traditional medicine as a laxative and diuretic agents and during the years were subjected to research and besides the aforementioned cucurbitacins (I, J, and T) [28], their glycosides, as well as, colocynthosides A (hydroxyl derivative of cucurbitacin E 2-O-β-D-glucopyranoside) and B (a derivative that liberated L-rhamnose and D-glucose after hydrolysis) [30] or ring-A-modified *seco*-cucurbitane triterpenoids were isolated [31].

Plants of the genera *Cucumis* and *Cucurbita* are widely cultivated and used for culinary purposes throughout the world. However, they are often not cucurbitacin-free plants and these substances have been identified and isolated from the following species: *Cucumis melo* [9,32], *C. prophetarum* [33], *C. sativus* [34], *Cucurbita andreana* (*C. maxima*) [35], and *C. pepo* var. *cylindrica* [36]. The biosynthesis of cucurbitacins is not fully understood, but it is assumed that drought and temperature may affect their production in plants and this issue will be addressed in the subsequent chapter. It was revealed that *Cucumis* plants are the main natural source of cucurbitacin A, which was isolated for instance from melon stems together with other cucurbitacins and their glucosides, the so-called arvenins I and III [32]. Another compound with limited natural occurrence is cucurbitacin C, which is mainly found in cucumber (*C. sativus*), and the authors described 10 new analogues of cucurbitacin C in cucumber leaves [34].

Another frequently studied plant species is *Ecballium elaterium*. This plant is abundant in the Mediterranean region and is also known as the “squirting cucumber”, whose fruit juice proved to be a remedy for jaundice in folk medicine [37,38]. The fruit juice was the source of cucurbitacins B, D, E, and I, their glycosylated forms and 22-deoxocucurbitacin D [37,38].

Cucurbitacins, their isoforms, acetyl, and dihydro derivatives were also isolated from the tubers of *Hemsleya ellipsoidea* [39], the roots of *Ibervillea sonorae* [40], the fruits of *Lagenaria siceraria* [41], *Luffa graveolense* [42], and *L. operculata* [18].

It turns out that the fruit extract of *Momordica charantia*, a bitter melon vine with the ability to lower blood glucose, contained several cucurbitane-type triterpene glycosides named after their Latin name—momordicosides A, B, K, L, M, N, and S, as well as, other compounds such as karavilosides II and III, or kuguaglycoside B named after the Mandarin Chinese word “kǔguā” that means “bitter melon” [43].

Finally, within the *Cucurbitaceae* family, the compounds described in this study were also isolated from the fruit methanol extract of *Sechium edule* var. *nigrum spinosum* [44], extracts from the roots and fruits of plants from the *Trichosanthes* genus, i.e., *T. cucumerina* [45], *T. kirilowii* [46,47], *T. tricuspidata* [48], or the roots of *Wilbrandia ebracteata* [17].

Cucurbitacins and their derivatives are also biosynthesized in many other plant families and fungi, and Table 2 summarizes the data from the last 50 years of research on the isolation of cucurbitacins and the identification of new derivatives of these compounds in organisms other than cucurbits.

These compounds have been found in 15 other plant families. They can be found in the plants of the *Begonia* genus, e.g., *B. heracleifolia* [49] and *B. nantoensis* [50], as well as, in *Iberis amara*, *I. gibraltarica*, and *Lepidium sativum* from *Brassicaceae* [51,52]. Cucurbitacins were also isolated from *Cercidiphyllum japonicum* [53], *Datisca glomerata* [54], *Desfontania spinosa* [55], or *Licania intrapetiolaris* [56]. They are also widespread in various species of *Elaeocarpus*, e.g., *E. hainanensis*, *E. mastersii*, or *E. sylvestris*, where cucurbitacins and their glycosides were identified [57,58,59]. In the same plant family, i.e., *Elaeocarpaceae*, cucurbitacin D, 2-deoxycucurbitacin D, and 25-acetylcucurbitacin F were isolated from *Sloanea zuliaensis* [60]. Moreover, it is easy to find producers of cucurbitacins from the well-known *Lauraceae* or *Malvaceae* families, i.e., *Machilus yaoshansis* [61,62] and *Helicteres isora* [63], respectively.

Interestingly, the described compounds are widely distributed in the *Plantaginaceae* family [64,65,66,67,68,69,70]. In most of them, the cucurbitacins were linked to different sugars. Those presented in *Bacopa monnieri* are so-called bacobitacins and in their structures arabinose and rhamnose sugar residues can be distinguished [64]. Scrophoside A was identified in the rhizomes of *Neopicrorhiza scrophulariiflora* [66], while picfeltarraenins IA, IB, IV, and VI (cucurbitane-type glycosides with xylose and rhamnose residues) were the active compounds of *Picria fel-terrae* extract [67]. In addition, several derivatives and glycosides of cucurbitacins were elucidated after their extraction from the roots of *Picrorhiza kurroa* [68,69] and *P. scrophulariiflora* [70].

In the search for plant anti-cancer agents, Arisawa et al. [71] focused on *Ipomopsis aggregata*, which was the source of cucurbitacin B, isocucurbitacin B, and 3-*epi*-isocucurbitacin B. In addition, the previously mentioned arvenins found in the *Cucumis* genus are cucurbitacin glucopyranosides and were named after *Anagallis arvensis* [72].

*Rosaceae* is another plant family worth mentioning due to the biosynthesis of cucurbitacins. Examples of plants from this family are *Kageneckia angustifolia* and *K. oblonga*, and from the seeds and the aerial parts, respectively, compounds such as cucurbitacin F, 2,3,16-triacetylcucurbitacin F, and 3β-(β-D-glucosyloxy)-16α,23α-epoxycucurbita-5,24-dien-11-one were isolated [73,74]. Cucurbitacins D and F, and their derivatives were also found in *Physocarpus capitatus* and *P. opulifolius* [75,76], as well as, in *Purshia mexicana* (*Cowania mexicana*) [77] and *Sorbaria sorbifolia* var. *stellipila* [78].

Finally, cucurbitacins, their isomers, were found in the aerial parts of *Nernstia mexicana* (*Cigarrilla Mexicana*) [80,81] from the *Rubiaceae* family and, within the *Thymelaeaceae* family, they were found in the leaves of *Aquilaria sinensis* [82] and in the twigs of *Gonystylus keithii* [83].

Unusually, work by Clericuzio et al. [12,13] showed that cucurbitacins can also be found in higher fungi (*Basidiomycetes*), and more specifically in *Leucopaxillus gentianeus* of the family *Tricholomataceae*. Cucurbitacin B is mainly responsible for the bitter taste of the mushroom flesh, but it also occurs in the fruiting bodies in the esterified tasteless form as the following compounds: oleyl, linoleyl, and palmityl esters of cucurbitacin B [12]. Furthermore, three novel cucurbitane triterpenoids, namely, leucopaxillones A and B, and 18-deoxyleucopaxillone A, as well as, the known cucurbitacin D and 16-deoxycucurbitacin B were isolated from the fruiting bodies or the mycelia of *L. gentianeus* [12,13].

## 3. Effect of Drought and Temperature on the Biosynthesis of Cucurbitacins

Both drought stress and temperature or insufficient light deficiency affect plant growth and can have a critical impact on plant metabolism. These factors inhibit numerous morphological, physiological, and biochemical processes, leading to a 30% loss by 2025 [84]. It turns out that the biosynthesis of cucurbitacins also depends on various environmental conditions, resulting in a bittering effect on plants and, in the case of those grown for food purposes, the deterioration of their taste reduces their marketability, ultimately leading to a decline in demand for the crop and a loss of profit for farmers [85]. However, there is still no well-documented explanation of the relation between the effects of abiotic stresses and the levels of the described chemical compounds in plants of the *Cucurbitaceae* family, but recent publications attempted to address this problem.

Shang et al. [86] discovered nine cucumber genes in the biosynthesis pathway of cucurbitacin C and two transcription factors *Bl* (Bitter leaf) and *Bt* (Bitter fruit), responsible for regulating of the biosynthesis pathway in leaves and fruits, respectively. The authors also showed that some cucumber fruits became bitter when the plants were grown at the temperature of 18 °C during the day, and 12 °C at night, while no bitterness was observed at temperature of 30 °C/22 °C (day/night). Other abiotic stresses also stimulated the biosynthesis of cucurbitacins and increased expressions of the *Bi* gene and *Csa5G156220* (*Bl* gene) were found in cucumber plants exposed to drought stress or treatment with abscisic acid [86].

In other studies, which were not conducted at the molecular level, it was found that the occurrence of bitter cucumber fruits in *Cucumis sativus* L. cv. Kagafutokyuri was more frequent when the plants were grown at lower temperatures and also when the plants were cultivated with doubled nitrogenous fertilizers [87]. In addition, cucurbitacin C, the main bitter compound in cucumbers, is synthesized from mevalonic acid via 3-hydroxy-3-methylglutaryl Coenzyme A (HMG-CoA). The activity of HMG-CoA reductase, an enzyme involved in the biosynthesis of cucurbitacin, was significantly higher in bitter fruits, resulting in increased production of this compound [87].

Metabolomic and transcriptomic analyses were also used to investigate the mechanisms underlying the biosynthesis of cucurbitacins in *Luffa acutangula*. The use of LC-ESI-MS/MS system allowed the authors to confirm that the concentration of cucurbitacins was significantly higher in bitter than non-bitter *Luffa* fruits [88]. The authors verified whether drought stress and abscisic acid treatment affected the cucurbitacin biosynthetic pathway. The transcriptome analyses performed by Zhao et al. [88] confirmed that both drought stress and phytohormone application significantly increased the expression of *Bi*, cytochromes P450s (*CYP450s*), and acyltransferase (*ACT*) genes, the three genes related to cucurbitacin production in *L. acutangula* [88].

**Figure 6:**
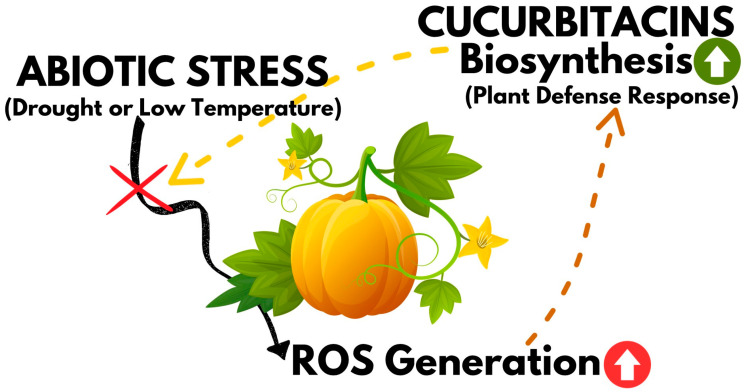
Graphical representation of cucurbitacin biosynthesis as a plant defence mechanism against abiotic stresses.

Mashilo et al. [89] studied the response of 12 landraces of bottle-gourd (*Lagenaria siceraria*) to drought stress, comparing various factors related to leaf gas exchange and chlorophyll fluorescence, and investigating cucurbitacin production. Cucurbitacins E and I were detected in both drought-stressed and non-stressed plants, but only the latter was strictly dependent on the experimental conditions. Furthermore, positive correlations were observed between cucurbitacin I concentration and electron transport to oxygen molecules (ETR/A) and alternative electron sink (AES). In addition, the authors proposed an explanation for this phenomenon, that cucurbitacin I may act as an antioxidant against oxidative stress caused by reactive oxygen species formed under drought stress [89]. Thus, it can be considered that the production of cucurbitacins is a plant defense response to unfavorable conditions, and the deterioration of fruit taste due to the bitterness of these compounds is, in a sense, a side effect of this response (Figure 6).

## 4. The Use of Cucurbitacins in Cancer Research

The anticancer activity of cucurbitacins has been demonstrated in the treatment of many cancers such as: breast, cervical, cholangiocarcinoma, colon, gastric, glioblastoma, hepatoma, lung, laryngeal, lymphoma, malignant glioma, melanoma, neuroblastoma, osteosarcoma, ovarian, pancreatic, prostate, and tongue, in many cases both in vitro and in vivo [8,40,90,91,92,93,94,95,96,97,98,99,100,101,102,103,104,105,106,107,108,109,110,111,112,113,114,115,116,117,118,119,120,121,122,123,124,125,126,127,128,129,130,131,132,133,134,135,136]. The details of selected studies on cucurbitacins and their anticancer activity with the mechanism of action are presented in Table 3.

### 4.1. Biological Activity and Breakthroughs on Cucurbitacin Efficacy

Cucurbitacins, exhibit many potent biological effects, and their potency varies depending on the specific target cells. These effects include cytotoxicity, anticancer properties, hepatoprotective properties [137], anti-inflammatory effects [138], antimicrobial defense [139], antiviral [140], and anthelmintic properties [3], cardiovascular benefits [141], antidiabetic and antihyperglycemic efficacy [142], and cardioprotective properties [141]. This spectrum of activities has been investigated both in vivo and in vitro, determining the therapeutic potential of the most commonly used cucurbitacins [3]. The primary functions of the reported secondary metabolites include anticancer activity, most commonly through induction of apoptosis, and regulation of cell proliferation [143]. Many studies, point to their potential as pioneers in pharmacological innovation, due to their proficient modulation of molecular mechanisms. These compounds are not only intriguing for their biochemical novelty, but also for their potential to transform the therapeutic arsenal in the ongoing battle to treat difficult cancers. Many studies are very promising, for example in targeted studies, such as in pancreatic cancer, where cucurbitacin B was shown to significantly reduce tumor size and volume in pancreatic cancer xenograft models, showing promise as a potent therapeutic agent [113]. Cucurbitacins also have potential in combination therapies. Recent research has delved into combining cucurbitacins with other treatments. For example, when combined with chemotherapeutic agents like cisplatin, cucurbitacins have been shown to increase apoptosis in ovarian cancer cells [144]. Also important is the possibility of using cucurbitacins in nanomedicine; cucurbitacin nanoparticles were developed to improve bioavailability and targeted delivery, maximizing therapeutic efficacy [145,146].

### 4.2. Mechanism of Action

**Figure 7:**
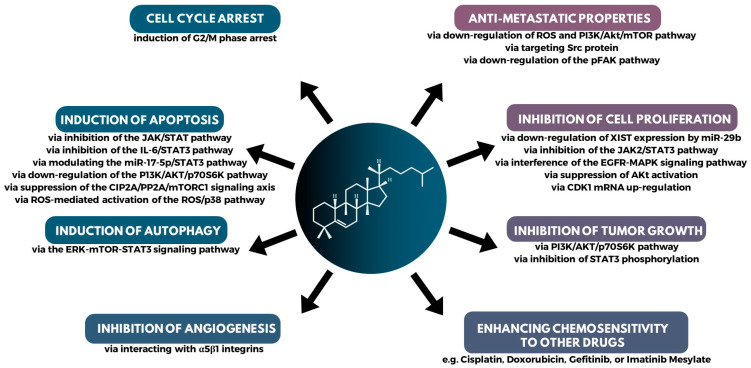
A summary of the major mechanisms of action in the anticancer activity of cucurbitacins.

The question arises: how do cucurbitacins fight cancer cells? Cucurbitacins owe their anticancer properties to multiple mechanisms of action. First and foremost, cucurbitacins have anticancer and anti-inflammatory properties. They exert anticancer effects through various mechanisms, such as inhibiting tumor cell proliferation, inducing cell cycle arrest and apoptosis (programmed cell death), inhibiting invasion and metastasis, and disrupting the protein backbone (Figure 7) [92,93,147]. Regarding proliferation inhibition, cucurbitacins exhibit antiproliferative activity in many cell lines, without significant cellular and tissue specificity [7]. In addition, it has been shown that they can, inhibit angiogenesis (the formation of new blood vessels that supply blood to the tumor) and affect the migration and invasion of tumor cells.

There are many studies connecting cucurbitacin with apoptosis. Apoptosis, or programmed cell death, is a mechanism by which damaged or old cells undergo self-destruction. In many cancers, this process is inhibited, leading to uncontrolled cell proliferation. Cucurbitacins restore the apoptotic pathway in cancer cells, activating pro-apoptotic proteins and inhibiting anti-apoptotic proteins. The result is the selective death of cancer cells, leaving healthy cells unharmed [148]. For example, cucurbitacin E (CuE) was able to inhibit the growth of human breast cancer cells in vitro. CuE induced both apoptosis and cell cycle arrest probably through the inhibition of Signal Transducer and Activator of Transcription (STAT3) function [125]. Similarly, cucurbitacin D inhibits cell growth and induces apoptosis through inhibition of STAT3 activity in breast cancer cells [149]. Cucurbitacins effectively inhibit the JAK2/STAT3 signalling pathway, down-regulating genes responsible for cell proliferation, survival, angiogenesis, and metastasis. By targeting this important pathway, cucurbitacins exhibit profound anti-tumor activities. STAT3 is an oncogenic transcription factor frequently activated in many types of cancer. Overactive STAT3 often leads to tumor progression, making it an important target in cancer therapy. Cucurbitacins can effectively inhibit STAT3 phosphorylation, thereby limiting its transcriptional activity and thus suppressing tumor growth [3,150]. The apoptotic mechanism effect of cucurbitacin B was also observed in the case of neuroblastoma via JAK2/STAT3 and MAPK pathway [109] and in osteosarcoma [110]. Apoptosis was also observed in action for CuB in cancer cells via inhibition of the IL-6/STAT3 pathway [104], via modulating the miR-17-5p/STAT 3 [106], and for CuI via down-regulation of the PI3K/AKT/p70S6K pathway [130] in lung cancer cells. The induction of apoptotic cell death by suppressing CIP2A/PP2A/mTORC1 in gastric cancer cell [99] and by mTOR/PI3K/Akt in ovarian cancer cells [91].

In the study by Üremiş et al. [151], which aimed to investigate the anticancer effect with hepatocellular carcinoma cells, it occured that cucurbitacin E down-regulated JAK2/STAT3, PI3K/Akt/mTOR, MAPK signalling pathway proteins, and Bcl-xL levels, while up-regulating Caspase-9 and Bax protein levels [151]. Thus, cucurbitacin E arrested the cell cycle in the G2/M phase while causing mitochondrial and DNA damage. The ability of cell cycle arrest was shown also for other cucurbitacins which arrest the cell cycle, particularly at the G2/M phase, thereby preventing the proliferation of malignant cells. By interfering with the regular progression of the cell cycle, these compounds prevent cancer cells from proliferating uncontrollably. Cucurbitacin B has been observed to arrest the cell cycle at the G2/M phase, effectively stopping the division and growth of malignant breast cancer cells [152]. The disruption of the cell cycle renders cancer cells more susceptible to the effects of chemotherapeutic agents [7]. Cucurbitacin D induces G2/M phase arrest and apoptosis via the ROS/p38 pathway in the Capan-1 pancreatic cancer cell line [153]. Lastly, it was found that cucurbitacin B substantially reduced the growth of conjunctival melanoma (CM) cells while being non-toxic to healthy cells and can also arrest cells at G2/M phase in CM cells [108]. The G2/M cell cycle arrest and mTOR/PI3K/AKT were observed in lung cancer cells [90], and in breast cancer via ATM mediated damage [93]. Anti-proliferation and anti-invasion effects via G2/M arrest was observed in prostate and colon cancer [8]. Cell cycle arrest was observed also in laryngeal squamous via inhibition of Bcl-2 and cyclin B1 [103] and in pancreatic cancer cells via downregulation of MUC13 and restoration of miR-145 expression [119]. There are many reports regarding the effect of cucurbitacins in inhibiting tumor cell growth and proliferation. Anti-proliferation was observed in squamous cell carcinoma of the tongue by downregulation of XIST via miR-29b [116] and by downregulation of JAK2/STAT3 in gastric cancer cells [154]. The growth inhibition by JAK2/STAT3 was observed in pancreatic cancer cells [134].

In Wu et al. [8] studies, it was shown that cell cycle arrest and apoptosis are present in lung cancer, together with antiproliferation and anti-metastasis [8].

Cucurbitacins can inhibit processes such as epithelial-mesenchymal transition, making the cancer cells less aggressive and reducing metastasis. An example of anti-metastatic potential of cucurbitacin B was shown in cholangiocarcinoma cells by targeting the Src protein [97]. Anti-metastasis via down-regulation of ROS (reactive oxygen species) and PI3/Akt/mTOR in lung cancer cells [9], and CuB via downregulation of the pFAK pathway in breast cancer [92].

There are also other reports of the involvement of cucurbitacins in the process of autophagy, which plays a role in the suppression of neovascularization. An example of this is cucurbitacin I, which induced autophagy through the ERK-mTOR-STAT3 pathway, in lung cancer cells [131], and cucurbitacin B induced DNA damage and ROS-mediated autophagy in breast cancer cells [155].

Cucurbitacins can be used to target Cancer Stem Cells (CSCs), a subset of cancer cells known to initiate tumorigenesis and contribute to drug resistance and cancer relapse. Cucurbitacins target these elusive CSCs, rendering them more vulnerable to treatment. Through modulating pathways such as the Wnt/β-catenin and Hedgehog signalling pathways, cucurbitacins impair the self-renewal capacities of CSCs, thereby reducing their population within tumors and reducing their potential threat [156].

Chronic inflammation plays a key role in the development of several cancers. Cucurbitacins, with their anti-inflammatory properties, inhibit the activation of NF-κB, a protein complex that plays a key role in regulating the immune response to infection. Cucurbitacin B and cucurbitacin E have been identified as the most investigated when it comes to the immune response, playing roles in both innate and adaptive immunity. The most cited mechanisms were inhibition of COX-2 and NOS, reduction of oxidative stress, suppression of proinflammatory cytokines and modulation of acquired immunity proteins [157]. By mitigating inflammatory pathways and modulating the tumor microenvironment, cucurbitacins can potentially prevent the early stages of tumorigenesis and also provide an avenue for combined therapeutic strategies with other drugs [157]. The details of selected studies on cucurbitacins and their efficacy, together with the mechanism of action are presented in Table 3.

### 4.3. Toxicity and Safety Dosage

While the therapeutic potential of cucurbitacins is evident, certain challenges need to be overcome, such as: (1) toxicity: high concentrations can induce toxicity in non-cancerous cells. It is therefore imperative to identify the optimal therapeutic window; (2) bioavailability: being hydrophobic, cucurbitacins have limited solubility in aqueous solutions, which hinders their bioavailability; (3) pharmacokinetic profiles: a comprehensive understanding of their metabolism, distribution, and excretion in the human body is essential for effective clinical application.

Cucurbitacins are known for their health-promoting properties, but studies also show that they can have toxic effects. The exact mechanisms underlying the toxicity of cucurbitacins are unknown. While some studies suggest that cucurbitacins inhibit certain enzymes, others suggest that they disrupt the integrity of cell membranes. This duality of mechanisms adds another layer of ambiguity to their toxicological profile. They interact with a wide range of cellular components, including cell surface receptors, transcription factors, and signalling pathways. This extensive repertoire of interactions adds to the complexity of explaining their biological effects. The toxicity of cucurbitacins is not uniform across cell or tissue types. Their effects vary depending on the specific context, which introduces an element of unpredictability. Some cells may succumb to cytotoxicity, while others may experience growth inhibition or apoptosis, which increases the severity of their toxicological profile, but in turn contributes to their anticancer effects. Elucidation of the substitution patterns of various cucurbitacins has played a key role in distinguishing between their toxic effects and potential therapeutic properties [11]. Cucurbitacins are well known for their highly toxic nature, as evidenced by severe poisonings and deaths of livestock consuming bitter fruits from the genera *Cucumis* and *Cucurbita*. In in vivo studies, the reported toxicity of cucurbitacins ranges from 2 to 12.5 mg/kg, although individual cases have indicated much higher levels of toxicity, such as cucurbitacin R at 375 mg/kg and 67 mg/kg [65]. It is worth noting that the presence of a double bond at C23 and an acetyl group at C25 have been identified as factors that increase the toxicity of cucurbitacins [11]. Some reports revealed acute toxicity of cucurbitacins to animals and the following values of LD50 were observed: 1.2 mg/kg in male mice and 2.0 mg/kg in female rats when cucurbitacin A was used, 1.0 mg/kg in mice for cucurbitacin B, and 2.0 mg/kg in mice for cucurbitacin E [158,159]. Despite their toxicity, cucurbitacins exhibit strong biological activity, albeit at doses approaching toxic levels, making them unsuitable as biological agents. The highly bitter taste of cucurbitacins should naturally discourage their consumption by humans, but cases of poisoning after ingestion of cucurbitaceous plants have been reported [160]. Death have occurred after ingestion of *Luffa cylindrica* fruit [161] and gastrointestinal symptoms have been associated with ingestion of bottle gourd containing cucurbitacin D [162].

Extensive evaluations of cucurbitacins C, D, E and I have clearly classified them as lethal compounds. Consequently, consumption of plants containing these specific cucurbitacins, such as species of the genera *Cucumis* and *Cucurbita*, should be avoided to prevent illness or death [163]. The manifestation of toxic symptoms varies depending on factors such as the animal species used in the experiments, the route of administration and the dose administered [164].

The determinations of a safe dose of cucurbitacins remains elusive. Their toxicity is highly dose-dependent, and even small amounts can have adverse effects. This narrow therapeutic window is a major challenge for potential clinical applications, and adds to the complexity of their use [11].

While cucurbitacins have been extensively studied in vitro and in animal models, the transition to human studies adds another layer of complexity. Inter-individual differences in metabolism and genetic predisposition add an unpredictable dimension to the evaluation of the safety and efficacy of cucurbitacins in humans [11].

An important part of preclinical and clinical research is pharmacokinetic studies, which aim to examine how the administered drug interacts with the body. The fate of chemical compounds administered to the organism is abbreviated as ADME, which exactly means Absorption, Distribution, Metabolism, and Excretion. To date, research related to the ADME analyses of cucurbitacins has been carried out primarily on rats. Sophisticated chromatographic methods of high- or ultra-performance liquid chromatography coupled with tandem mass spectrometry served for the analysis of rat plasma samples [165]. In 1998, cucurbitacin tablets were approved by the China Food and Drug Administration (CFDA) as a treatment for chronic hepatitis and primary liver cancer [166]. The mentioned preparations with the content of 57 μg for cucurbitacin B and 19 μg for cucurbitacin E per tablet were administered orally to 10 male rats, and the authors observed double peaks in curves of mean plasma concentration-time profiles of both cucurbitacins, explaining this phenomenon through the distribution, reabsorption, and enterohepatic circulation [166]. The low absolute bioavailability of cucurbitacin B (1.37%) was noted after oral administration in Wistar rats, where the solubility and membrane permeability played a key role in reaching the systemic circulation, hence the form of the preparation needs improvements in order to enhance its bioavailability [167]. Compared to oral administration, when cucurbitacin B was administered intravenously to rats, the time to reach the maximum concentration (T_max_) was 25 times shorter and amounted to approximately 7 min, which indicated rapid absorption [167]. In addition, Hunsakunachai et al. [168] showed also a wide distribution of cucurbitacin B in various tissues, especially lung, spleen, and kidney tissues with a high tissue-to-plasma concentration ratio (K_app_). Moreover, chromatographic and spectroscopic techniques acknowledged how cucurbitacin B was metabolized. Tang et al. [169] revealed that it was metabolized to cucurbitacin D (a non-esterified version of the former). In contrast, Hunsakunachai et al. [168] showed that glucuronide conjugation took part in the metabolism of cucurbitacins.

The transition of any compound from the research phase to the pharmaceutical market depends on many factors, ranging from effectiveness and safety to production capacity and market dynamics. Although the activity of cucurbitacins against cancer cells is promising, there are challenges affecting pharmaceutical progress such as toxicity, pharmacokinetics, bioavailability, and delivery systems. Some cucurbitacins exhibit rapid metabolism and excretion, which may limit their therapeutic utility. Further research on these compounds, with particular emphasis on improving their therapeutic index and overcoming formulation and delivery challenges, may pave the way for their future inclusion in therapy.

### 4.4. Combination Therapy of Cucurbitacins and Other Drugs in Combating Cancer

Although cucurbitacins have anticancer properties, several studies evaluated a combination therapy of cucurbitacins with other commonly used chemoterapeutic agents where their synergy may improve treatment efficacy, and reduce cancer cell resistance [7,170]. The latter was confirmed when cucurbitacin I at the concentration of 100 nM sensitized the colon cancer cell line COLO205 to 5-fluorouracil treatment. The authors also demonstrated that cucurbitacin I inhibited cell migration and invasion in vitro [128]. In addition to 5-fluorouracil, another nucleoside analogue, gemcitabine, has been used in synergism studies [94,114]. Put simply, both compounds lead to the inhibition of DNA synthesis and subsequent cell death. Thoennissen et al. [114] demonstrated a synergistic antiproliferative effect of cucurbitacin B-gemcitabine on pancreatic cancer cells. Then, in the in vitro studies by Aribi et al. [94], cucurbitacin B in combination with gemcitabine inhibited the proliferation of MDA-MB-231 breast cancer cells and remarkably reduced the tumor volume compared to the with the monotherapies of the tested chemicals. The same observation was made for the combination of cucurbitacin B and docetaxel, a semi-synthetic analog of paclitaxel [94]. The latter, i.e., paclitaxel, sold under the brand name “Taxol” was isolated from the bark of *Taxus brevifolia*. Combined treatment with 2-deoxy-2-amine-cucurbitacin E (a semisynthetic derivative of cucurbitacin B) and paclitaxel showed high capability in for growth inhibition and proliferation of the A549 human non-small cell lung cancer (NSCLC) cell line, where NSCLC has a high mortality rate and is one of the most common malignant tumors. Importantly, the use of cucurbitacin derivative and paclitaxel did not result in liver and kidney tissue damage [171].

Cisplatin, PtCl_2_(NH_3_)_2_, is a cytostatic chemotherapy drug used in the treatment of several types of malignant tumors. This inorganic chemical compound and planar coordination complex was also investigated in the combination therapy either with cucurbitacins B orE. Platinum-based drugs are among the first line treatments for ovarian cancer, the most deadly gynecological cancer. However, the emergence of cisplatin-resistant tumor has led to the need for alternative therapies. According to El-Senduny et al. [112], cucurbitacin B showed cytotoxicity against the ovarian cancer cell lines and has been defined as a chemosensitizer for cisplatin-resistant cell lines. Futhermore, the combination of cucurbitacin B and cisplatin had a synergistic effect on the induction of apoptosis, cell cycle arrest, and growth inhibition of Hep-2 laryngeal cells [172]. In addition, cucurbitacin E enhanced the growth inhibition of human breast cancer cells, when cisplatin was used. Cucurbitacin E affected G2/M phase arrest and cell apoptosis by inhibiting STAT3 function [125].

Other inorganic compounds used in cancer treatment include, e.g., arsenic trioxide (As_2_O_3_). Similar to the above work, inhibition of STAT3 phosphorylation was also observed in Burkitt’s lymphoma Ramos cells when cucurbitacin B was combined with arsenic trioxide [107].

Doxorubicin is an anthracycline compound with cytostatic activity widely used in cancer treatment, whose mechanism of action is based on interaction with DNA by intercalation, leading to cell death. Its use in combination with selected cucurbitacins has also been tested on various cancer cell lines. In human anaplastic thyroid carcinoma cells, cotreatment of doxorubicin and cucurbitacin B was applied resulting in increased cytotoxic activity compared to doxorubicin alone, whereas their synergistic cytotoxicity was mediated by, e.g., survivin, BCL-2 family proteins, ROS, and the JAK/STAT signalling pathway [173]. In another study, cucurbitacin D and doxorubicin were examined in MCF7/ADR breast cancer cells. Co-application of cucurbitacin D decreased cell proliferation, induced apoptosis, and G2/M cell cycle arrest by inhibiting STAT3 and NF-κB signalling [117]. Cucurbitacin E was also able to act in a similar way to other cucurbitacins when used in combination with doxorubicin against gastric cancer cell lines. Both in vitro and in vivo experiments confirmed the synergistic effect of these compounds, and cucurbitacin E significantly enhanced the cytotoxic properties of doxorubicin [126]. The study of Sadzuka et al. [133] used cucurbitacin I, which is thought to have antioxidant properties. The combination of cucurbitacin I and doxorubicin was characterised by increased activity against M5076 ovarian sarcoma cells. Moreover, co-administration of these compounds also reduced tumor size and weight in mice. The antioxidant activity was shown to be critical in reducing cardiac damage and suppressing the associated doxorubicin-induced lipid peroxide production in the heart [133].

Over the last dozen years, cucurbitacins B, E, D, and I were evaluated for synergistic effects with other molecules. Jing et al. [7] and Garg et al. [170], in their review articles presented papers investigating the combination of cucurbitacins with the following compounds: valproic acid, cerulenin, bortezomib, irinotecan, methotrexate, imatinib mesylate or gefitinib. Depending on the studies performed (in vitro vs. in vivo), scientists showed that the use of cucurbitacins as an adjunct substance in cancer treatment led to the induction of apoptosis, inhibition of cell growth and proliferation, and cell cycle arrest. Cucurbitacins also increased the sensitivity of other molecules and were able to reduce tumor volume [7,170].

### 4.5. Future Directions: Paving the Path Forward

The use of cucurbitacins in cancer therapy is still in its early stages. However, the pace of research, as evidenced by the increasing number of published reports (Figure 2), suggests a promising future. Applications in personalized medicine could be considered, as advances in genomics and proteomics will make it possible to tailor cucurbitacin-based therapies to individual patient profiles. It is also worthwhile to expand research into the spectrum of action of cucurbitacins. Current research is mainly focused on specific types of cancer. There is a need to investigate the efficacy of cucurbitacins in a wider range of malignancies. It is becoming increasingly clear that these compounds with their potent anticancer properties have the potential to change the landscape of oncology as our scientific efforts progress. While the therapeutic potential of cucurbitacins is undeniable, applying them is challenging. Careful formulation and dosing strategies are required due to their low water solubility and potential toxicity at high doses. Recent advances in nanotechnology offer potential solutions, enabling targeted drug delivery and improved solubility. In addition, the combination of cucurbitacins with other anticancer agents can provide synergistic effects. This maximizes therapeutic outcomes while minimizing side effects.

## 5. Conclusion: The Rising Star of Botanical Therapeutics

The study of cucurbitacins underscores the profound wisdom embedded in nature. It is becoming increasingly clear that these compounds, with their potent anticancer properties, have the potential to reshape aspects of oncology as we advance in our scientific endeavors. Through collaborative research, innovative methodologies, and unwavering dedication, we may soon be ushered in an era in which cucurbitacins will become an integral part of cancer therapeutics. Cucurbitacins in the fight against cancer offer multiple mechanisms to target the multifaceted nature of the disease. They could be incorporated into the next generation of anticancer drugs, offering hope to millions of people, if we continue to harness and challenge their power. To support development towards upcoming clinical trials, the cucurbitacins must first demonstrate the efficacy of their anticancer properties in mouse models of xenograft tumors. Cucurbitacins are a potential class of drugs that can be used to enhance the efficacy of existing anticancer therapies. The addition of cucurbitacins to chemotherapy or radiotherapy significantly increases anticancer efficacy. The bioavailability and stability of cucurbitacins will be increased by studying metabolic changes. Cucurbitacins may soon be used in the treatment of various diseases, including chronic diseases and cancer. This is due to their low level of toxicity and high level of efficacy. Current research provides a solid foundation for future research and development in the discovery of safer and more effective anticancer drugs.

## Figures and Tables

**Table 1:** Examples of cucurbitacins found in plant of the *Cucurbitaceae* family.

Plant	Present Cucurbitacins
*Acanthosicyos horridus*	Cucurbitacins B and D, 23,24-dihydrocucurbitacin D (cucurbitacin R), 3-*epi*-isocucurbitacin D [20]
*Alsomitra macrocarpa*	Cucurbitacin E [21]
*Bolbostemma paniculatum*	Isocucurbitacin B, 23,24-dihydroisocucurbitacin B, cucurbitacin E, 23,24-dihydrocucurbitacin E [22]
*Bryonia cretica*	Cucurbitacins G, H, and J, and isocucurbitacins G and H [23]; Cucurbitacins B, D, E, and J, 23,24-dihydrocucurbitacins B and E, bryoniaosides A and B, and hexanocucurbitacin D [24]
*Cayaponia racemosa*	Cucurbitacin P, 11-deoxocucurbitacin P, deacetylpicracin [25] cucurbitacins B, D, and F, 23,24-dihydrocucurbitacins F and D (cucurbitacin R) [26]
*Cayaponia tayuya*	Cucurbitacin R and 23,24-dihydrocucurbitacin B [27]
*Citrullus colocynthis*	Cucurbitacins I, J, and T [28]; cucurbitacin B 2-*O*-β-D-glucopyranoside, cucurbitacin E 2-*O*-β-D-glucopyranoside [29]; 2-*O*-β-D-glucopyranosides of cucurbitacin E, I, J, K and L, and other derivatives [30]; ring-A-modified *seco*-cucurbitane triterpenoids [31]
*Cucumis melo*	Cucurbitacins B, D, and E [9]; cucurbitacins A, B, G, H, and R, 23,24-dihydrocucurbitacin B, 23,24-dihydroisocucurbitacin B, isocucurbitacin R, hexanorcucurbitacin D, arvenin I (cucurbitacin B 2-*O*-β-D-glucopyranoside), arvenin III (cucurbitacin D 2-*O*-β-D-glucopyranoside), 19-norlanosta-5,24-dien-11-one [32]
*Cucumis prophetarum*	Cucurbitacins B, E, I, and Q, isocucurbitacins B and D, 23,24-dihydrocucurbitacins B, E, I, and Q, 23,24-dihydroisocucurbitacin D [33]
*Cucumis sativus*	Cucurbitacin C and its derivatives [34]
*Cucurbita andreana*/ *Cucurbita maxima*	Cucurbitacins B, D, E, and I, 2-*O*-β-D-glucopyranosides of cucurbitacin B, E, and I [35]
*Cucurbita pepo* var. *cylindrica*	Cucurbitacin E 2-*O*-β-D-glucopyranoside [36]
*Ecballium elaterium*	Cucurbitacins B, D, E, and I [37]; Cucurbitacin D and 22-deoxocucurbitacin D [38]
*Hemsleya ellipsoidea*	Isocucurbitacin B, 23,24-dihydroisocucurbitacin B, cucurbitacins F, 25-*O*-acetylcucurbitacin F, 25-*O*-acetyl-23,24-dihydrocucurbitacin F [39]
*Ibervillea sonorae*	23,24-Dihydrocucurbitacin F [40]
*Lagenaria siceraria*	Cucurbitacin I [41]
*Luffa graveolense*	Cucurbitacins D and E, isocucurbitacin B, cucurbitacin D 2-*O*-β-D-glucopyranoside, cucurbitacin B 20-*O*-β-D-glucopyranoside [42]
*Luffa operculata*	Cucurbitacins B, D, and E, isocucurbitacin B, neocucurbitacins A and B [18]
*Momordica charantia*	Momordicosides A, B, K, L, M, N, and S, karavilosides II and III, kuguaglycoside B and other derivatives [43]
*Sechium edule* var. *nigrum spinosum*	Cucurbitacins B, D, and I [44]
*Trichosanthes cucumerina*	Cucurbitacin B, 23,24-dihydrocucurbitacin B, bryonolic acid, bryononic acid [45]
*Trichosanthes kirilowii*	Cucurbitacins B and D, isocucurbitacins B and D, 3-*epi*-isocucurbitacin B, 23,24-dihydrocucurbitacins B and E, 23,24-dihydroisocucurbitacin B [46]; Cucurbitacin D, 23,24-dihydrocucurbitacin D [47]
*Trichosanthes tricuspidata*	2-*O*-β-D-glucopyranosides of cucurbitacine J, K, L and 25-*O*-acetylcucurbitacin L 2-*O*-β-D-glucopyranoside, khekadaengosides A–N [48]
*Wilbrandia ebracteata*	Cucurbitacins B, D, E, G, H, J, K, P and R, 23,24-dihydrocucurbitacins B and E, 23,24-dihydroisocucurbitacin B, 22-deoxocucurbitacin D, 3-*epi*-isocucurbitacin G, (20*R*)-25-acetoxy-3,16α,20-trihydroxy-30-*nor*-2-(β-D-glucopyranosyloxy)-1,2,3,4,5,10-dehydrocucurbit-6-ene-11,22-dione [17]

**Table 2:** Sources of cucurbitacins found in other families.

Family Name	Plant Name	Present Cucurbitacins
*Begoniaceae*	*Begonia heracleifolia*	Cucurbitacins B and D, and their 2-*O*-β-D-glucopyranosides, 23,24-dihydrocucurbitacins F and D (cucurbitacin R) [49]
	*Begonia nantoensis*	Cucurbitacins B, E, I, and 23,24-dihydrocucurbitacins B and E [50]
*Brassicaceae*	*Iberis amara*	Cucurbitacins E and I, and their 2-*O*-β-D-glucopyranosides [51]
	*Iberis gibraltarica*	Cucurbitacins D, E and I [52]
	*Lepidium sativum*	Cucurbitacin I [52]
*Cercidiphyllaceae*	*Cercidiphyllum japonicum*	Cucurbitacin D [53]
*Datiscaceae*	*Datisca glomerata*	Cucurbitacins B, D, F and cucurbitacin glycosides (datisosides B, C, D, E, F, G, H) [54]
*Columelliaceae*	*Desfontania spinosa*	23,24-dihydro-11-deoxocucurbitacin I, and spinosides A and B [55]
*Chrysobalanaceae*	*Licania intrapetiolaris*	Cucurbitacin B [56]
*Elaeocarpaceae*	*Elaeocarpus hainanensis*	Cucurbitacins D, F, G, I, O, 3-*epi*-isocucurbitacin G, and other derivatives [57]
	*Elaeocarpus mastersii*	Cucurbitacins D and F [58]
	*Elaeocarpus sylvestris*	Cucurbitacin D and mogroside derivatives [59]
	*Sloanea zuliaensis*	Cucurbitacin D, 2-deoxycucurbitacin D, and 25-acetylcucurbitacin F [60]
*Lauraceae*	*Machilus yaoshansis*	Machilaminosides A and B (derivatives of cucurbitacin I) [61]; 2-*O*-β-D-glucopyranosides of cucurbitacin I, J, and K and their derivatives [62]
*Malvaceae*	*Helicteres isora*	Cucurbitacin B and isocucurbitacin B [63]
*Plantaginaceae*	*Bacopa monnieri*	Bacobitacins A, B, C, D and cucurbitacin E [64]
	*Conobea scoparioides*	Cucurbitacin E [65]
	*Neopicrorhiza scrophulariiflora*	Scrophoside A, cucurbitacin glycosides [66]
	*Picria fel-terrae*	Picfeltarraenin IA, picfeltarraenin IB, picfeltarraenin IV, and picfeltarraenin VI [67]
	*Picrorhiza kurroa*	Cucurbitacin B, 23,24-dihydrocucurbitacin B, and various derivatives and glycosides of cucurbitacin B [68,69]
	*Picrorhiza scrophulariiflora*	2-*O*-β-D-glucopyranosyl-3,16,20, 25-tetrahydroxy-9-methyl-19-norlanosta-5, 23-diene-22-one, 2-*O*-β-D-glucopyranosyl-3,16, 20-trihydroxy-25-acetoxy-9-methyl-19-norlanosta-5, 23-diene-22-one, 2-*O*-β-D-glucopyranosyl-4,4,9, 14-tetramethyl-19-norpregn-5-en-20-one [70]
*Polemoniaceae*	*Ipomopsis aggregata*	Cucurbitacin B, isocucurbitacin B, and 3-*epi*-isocucurbitacin B [71]
*Primulaceae*	*Anagallis arvensis*	Arvenin I (cucurbitacin B 2-*O*-β-D-glucopyranoside), arvenin II (23,24-dihydrocucurbitacin B 2-*O*-β-D-glucopyranoside), arvenin III (cucurbitacin D 2-*O*-β-D-glucopyranoside), arvenin IV (cucurbitacin R 2-*O*-β-D-glucopyranoside) [72]
*Rosaceae*	*Kageneckia angustifolia*	Cucurbitacin F, 2,3,16-triacetylcucurbitacin F [73]
	*Kageneckia oblonga*	3β-(β-D-glucosyloxy)-16α,23α-epoxycucurbita-5,24-dien-11-one [74]
	*Physocarpus capitatus*	Cucurbitacin F, dihydrocucurbitacin F, and hydroxyl/acetyl derivatives of cucurbitacin F [75]
	*Physocarpus opulifolius*	Cucurbitacin D, cucurbitacin F, and 3-*epi*-isocucurbitacin D [76]
	*Purshia mexicana (Cowania mexicana)*	Cucurbitacin F, 23,24-dihydrocucurbitacin F, 15-oxo-cucurbitacin F, 15-oxo-23,24-dihydrocucurbitacin F [77]
	*Sorbaria sorbifolia* var. *stellipila*	Cucurbitacins D and F [78]
*Rubiaceae*	*Hintonia standleyana*	23,24-dihydrocucurbitacin F 3-*O*-β-D-glucopyranoside [79]
	*Nernstia mexicana* (*Cigarrilla mexicana*)	Cucurbitacin E, isocucurbitacin B, *epi*-isocucurbitacin B [80]; arvenin I (cucurbitacin B 2-*O*-β-D-glucopyranoside) [81]
*Thymelaeaceae*	*Aquilaria sinensis*	Cucurbitacin I 2-*O*-β-D-glucopyranoside, bryoamaride (Cucurbitacin L 2-*O*-β-D-glucopyranoside) [82]
	*Gonystylus keithii*	Cucurbitacins B and D [83]
*Tricholomataceae* (Fungi)	*Leucopaxillus gentianeus*	Cucurbitacin B, oleyl, linoleyl and palmityl esters of cucurbitacin B, leucopaxillones A and B [12]; cucurbitacin D, 16-deoxycucurbitacin B, 18-deoxyleucopaxillone A [13]

**Table 3:** The anticancer activity of cucurbitacins.

Cucurbitacin Type	Cancer Type	Mechanism	References
Cucurbitacin A	lung	cell cycle arrest	[90]
Cucurbitacin A	ovarian	induce apoptosis, cell cycle arrest	[91]
Cucurbitacin B	breast	anti-metastatic	[92]
Cucurbitacin B	breast	cell cycle arrested	[93]
Cucurbitacin B	breast	induce apoptosis, inhibit cell migration	[94]
Cucurbitacin B	breast	induce apoptosis, cell cycle arrest	[95]
Cucurbitacin B	breast	inhibit cell proliferation, induce apoptosis, cell cycle arrest	[96]
Cucurbitacin B	cholangiocarcinoma	anti-metastatic	[97]
Cucurbitacin B	colon	inhibit cell growth, cell cycle arrest	[98]
Cucurbitacin B	colon	inhibit cell proliferation, induce apoptosis	[97]
Cucurbitacin B	gastric	induce apoptosis	[99]
Cucurbitacin B	glioblastoma	induce apoptosis	[100]
Cucurbitacin B	hepatoma	induce apoptosis	[101]
Cucurbitacin B	glioblastoma	inhibit angiogenesis	[102]
Cucurbitacin B	laryngeal squamous	cell cycle arrest	[103]
Cucurbitacin B	lung	induce apoptosis	[104]
Cucurbitacin B	lung	anti-metastatic	[105]
Cucurbitacin B	lung	induce apoptosis	[106]
Cucurbitacin B	lymphoma	induce apoptosis	[107]
Cucurbitacin B	melanoma	cell cycle arrest	[108]
Cucurbitacin B	neuroblastoma	induce apoptosis	[109]
Cucurbitacin B	osteosarcoma	induce apoptosis	[110]
Cucurbitacin B	osteosarcoma	induce apoptosis, cell cycle arrest	[111]
Cucurbitacin B	ovarian	increase the sensitivity of cisplatin	[112]
Cucurbitacin B	pancreatic	induce apoptosis	[113]
Cucurbitacin B	pancreatic	inhibit cell proliferation	[114]
Cucurbitacin B	pancreatic	inhibit cell proliferation	[115]
Cucurbitacin B	tongue squamous	inhibit cell proliferation	[116]
Cucurbitacin C	colon	inhibition cell growth, cell cycle arrest, induce apoptosis	[8]
Cucurbitacin C	lung	inhibit cell proliferation, induce apoptosis, cell cycle arrest	[8]
Cucurbitacin C	prostate	inhibit cell proliferation	[8]
Cucurbitacin D	breast	inhibit cell proliferation, induce apoptosis	[117]
Cucurbitacin D	cervical	inhibit cells growth and metastasis, cell cycle arrest	[118]
Cucurbitacin D	pancreatic	cell cycle arrest	[119]
Cucurbitacin D	pancreatic	induce apoptosis, cell cycle arrest	[112]
Cucurbitacin D	prostate	inhibit cell growth	[120]
Cucurbitacin D	lung	cell cycle arrest	[121]
Cucurbitacin E	glioblastoma	inhibit cell proliferation, induce apoptosis, cell cycle arrest	[122]
Cucurbitacin E	lung	induce apoptosis, cell cycle arrest	[123]
Cucurbitacin E	malignant glioma	antiproliferative, inhibit cell growth, cell cycle arrest	[124]
Cucurbitacin E	breast	inhibit cell growth	[125]
Cucurbitacin E	gastric	enhance the cytotoxicity of DOX in cells	[126]
Cucurbitacin I	colon	inhibit cell proliferation	[127]
Cucurbitacin I	colon	inhibit cell migration	[128]
Cucurbitacin I	colon	induce apoptosis, inhibit cell proliferation, cell cycle arrest	[129]
Cucurbitacin I	lung	induce apoptosis	[130]
Cucurbitacin I	lung	autophagy induction	[131]
Cucurbitacin I	ovarian	induce apoptosis, autophagy induction	[132]
Cucurbitacin I	ovarian	inhibit cell growth	[133]
Cucurbitacin I	pancreatic	inhibit cell growth	[134]
Cucurbitacin I	pancreatic	induce apoptosis, induce autophagy	[132]
Cucurbitacin IIa (25-*O*-acetyl-23,24-dihydrocucurbitacin F)	lung	induce apoptosis, cell cycle arrest	[135]
Cucurbitacin IIb (23,24-dihydrocucurbitacin F)	lung	induce apoptosis, cell cycle arrest	[136]
	lung	inhibit cell growth, induce apoptosis	[40]

## References

[1] Varela C., Melim C., Neves B.G., Sharifi-Rad J., Calina D., Mamurova A., Cabral C. (2022). Cucurbitacins as potential anticancer agents: New insights on molecular mechanisms. J. Transl. Med..

[2] Zhou Y., Ma Y., Zeng J., Duan L., Xue X., Wang H., Lin T., Liu Z., Zeng K., Zhong Y. (2016). Convergence and divergence of bitterness biosynthesis and regulation in *Cucurbitaceae*. Nat. Plants.

[3] Alghasham A.A. (2013). Cucurbitacins—A Promising Target for Cancer Therapy. Int. J. Health Sci..

[4] Siegel R.L., Miller K.D., Fuchs H.E., Jemal A. (2022). Cancer statistics. CA Cancer J. Clin..

[5] Cronin K.A., Scott S., Firth A.U., Sung H., Henley S.J., Sherman R.L., Siegel R.L., Anderson R.N., Kohler B.A., Benard V.B. (2022). Annual report to the nation on the status of cancer, part 1: National cancer statistics. Cancer.

[6] Sung H., Ferlay J., Siegel R.L., Laversanne M., Soerjomataram I., Jemal A., Bray F. (2021). Global Cancer Statistics 2020: GLOBOCAN Estimates of Incidence and Mortality Worldwide for 36 Cancers in 185 Countries. CA Cancer J. Clin..

[7] Jing S., Zou H., Wu Z., Ren L., Zhang T., Zhang J., Wei Z. (2020). Cucurbitacins: Bioactivities and synergistic effect with small-molecule drugs. J. Funct. Foods.

[8] Wu D., Wang Z., Lin M., Shang Y., Wang F., Zhou J.Y., Wang F., Zhang X., Luo X., Huang W. (2019). In Vitro and In Vivo Antitumor Activity of Cucurbitacin C, a Novel Natural Product From Cucumber. Front. Pharmacol..

[9] Yuan R.Q., Qian L., Yun W.J., Cui X.H., Lv G.X., Tang W.Q., Cao R.C., Xu H. (2019). Cucurbitacins extracted from *Cucumis melo* L. (CuEC) exert a hypotensive effect via regulating vascular tone. Hypertens. Res..

[10] Chen C.H., Kuo T.C.Y., Yang M.H., Chien T.Y., Chu M.J., Huang L.C., Chen C.Y., Lo H.F., Jeng S.T., Chen L.F.O. (2014). Identification of cucurbitacins and assembly of a draft genome for *Aquilaria agallocha*. BMC Genom..

[11] Kaushik U., Aeri V., Mir S.R. (2015). Cucurbitacins—An insight into medicinal leads from nature. Pharmacogn. Rev..

[12] Clericuzio M., Mella M., Vita-Finzi P., Zema M., Vidari G. (2004). Cucurbitane Triterpenoids from *Leucopaxillus gentianeus*. J. Nat. Prod..

[13] Clericuzio M., Tabasso S., Bianco M.A., Pratesi G., Beretta G., Tinelli S., Zunino F., Vidari G. (2006). Cucurbitane Triterpenes from the Fruiting Bodies and Cultivated Mycelia of *Leucopaxillus gentianeus*. J. Nat. Prod..

[14] Enslin P.R. (1954). Bitter principles of the cucurbitaceae. I.—Observations on the chemistry of cucurbitacin A. J. Sci. Food Agric..

[15] Rolnik A., Olas B. (2020). Vegetables from the Cucurbitaceae family and their products: Positive effect on human health. Nutrition.

[16] Chen J.C., Chiu M.H., Nie R.L., Cordell G.A., Qiu S.X. (2005). Cucurbitacins and cucurbitane glycosides: Structures and biological activities. Nat. Prod. Rep..

[17] Farias M.R., Schenkel E.P., Mayer R., Rucker G. (1993). Cucurbitacins as Constituents of *Wilbrandia ebracteata*. Planta Med..

[18] Kawahara N., Kurata A., Hakamatsuka T., Sekita S., Satake M. (2001). Two Novel Cucurbitacins, Neocucurbitacins A and B, from the Brazilian Folk Medicine “Buchinha” (*Luffa operculata*) and Their Effect on PEBP2αA and OCIF Gene Expression in a Human Osteoblast-Like Saos-2 Cell Line. Chem. Pharm. Bull..

[19] Salehi B., Capanoglu E., Adrar N., Catalkaya G., Shaheen S., Jaffer M., Giri L., Suyal R., Jugran A.K., Calina D. (2019). Cucurbits Plants: A Key Emphasis to Its Pharmacological Potential. Molecules.

[20] Hylands P.J., Magd M.S. (1986). Cucurbitacins from *Acanthosicyos horridus*. Phytochemistry.

[21] Momma K., Masuzawa Y., Nakai N., Chujo M., Murakami A., Kioka N., Kiyama Y., Akita T., Nagao M. (2008). Direct interaction of Cucurbitacin E isolated from *Alsomitra macrocarpa* to actin filament. Cytotechnology.

[22] Tang Y., Li W., Cao J., Li W., Zhao Y. (2015). Bioassay-guided isolation and identification of cytotoxic compounds from *Bolbostemma paniculatum*. J. Ethnopharmacol..

[23] Sallam A.A., Hitotsuyanagi Y., Mansour E.S.S., Ahmed A.F., Gedara S., Fukaya H., Takeya K. (2010). Cucurbitacins from *Bryonia cretica*. Phytochem. Lett..

[24] Matsuda H., Nakashima S., Abdel-Halim O.B., Morikawa T., Yoshikawa M. (2010). Cucurbitane-type triterpenes with anti-proliferative effects on U937 cells from an egyptian natural medicine, *Bryonia cretica*: Structures of new triterpene glycosides, bryoniaosides A and B. Chem. Pharm. Bull..

[25] Dantas I.N.F., Gadelha G.C.M., Chaves D.C., Monte F.J.Q., Pessoa C., de Moraes M.O., Costa-Lotufo L.V. (2006). Studies on the Cytotoxicity of Cucurbitacins Isolated from *Cayaponia racemosa* (*Cucurbitaceae*). Z. Naturforsch..

[26] Jacobs H., Singh T., Reynolds W.F., McLean S. (1990). Isolation and ^13^C-NMR Assignments of Cucurbitacins from *Cayaponia Angustiloba*, *Cayaponia racemosa*, and *Guranias ubumbellata*. J. Nat. Prod..

[27] Recio M.C., Prieto M., Bonucelli M., Orsi C., Manez S., Giner R.M., Cerda-Nicolas M., Rios J.L. (2004). Anti-inflammatory activity of two cucurbitacins isolated from *Cayaponia tayuya* roots. Planta Med..

[28] Gamlath C.B., Gunatilaka A.A.L., Alvi K.A., ur Rahman A., Balasubramaniam S. (1988). Cucurbitacins of *Colocynthis vulgaris*. Phytochemistry.

[29] Tannin-Spitz T., Grossman S., Dovrat S., Gottlieb H.E., Bergman M. (2007). Growth inhibitory activity of cucurbitacin glucosides isolated from *Citrullus colocynthis* on human breast cancer cells. Biochem. Pharmacol..

[30] Yoshikawa M., Morikawa T., Kobayashi H., Nakamura A., Matsuhira K., Nakamura S., Matsuda H. (2007). Bioactive saponins and glycosides. XXVII. Structures of new cucurbitane-type triterpene glycosides and antiallergic constituents from *Citrullus colocynthis*. Chem. Pharm. Bull..

[31] Liu Y., Chen G., Chen X., Chen S.X., Gan L.S., Yuan T. (2018). Colocynthenins A-D, Ring-A *seco*-Cucurbitane Triterpenoids from the Fruits of *Citrullus colocynthis*. J. Nat. Prod..

[32] Chen C., Qiang S., Lou L., Zhao W. (2009). Cucurbitane-type triterpenoids from the stems of *Cucumis melo*. J. Nat. Prod..

[33] Afifi M.S., Ross S.A., ElSohly M.A., Naeem Z.E., Halaweish F.T. (1999). Cucurbitacins of *Cucumis prophetarum* and *Cucumis prophetarum*. J. Chem. Ecol..

[34] Qing Z., Shi Y., Han L., Li P., Zha Z., Liu C., Liu X., Huang P., Liu Y., Tang Q. (2022). Identification of seven undescribed cucurbitacins in *Cucumis sativus* (cucumber) and their cytotoxic activity. Phytochemistry.

[35] Halaweish F.T., Tallamy D.W. (1993). A new cucurbitacin profile for *Cucurbita andreana*: A candidate for cucurbitacin tissue culture. J. Chem. Ecol..

[36] Hutt T.F., Herrington M.E. (1985). The determination of bitter principles in zucchinis. J. Sci. Food Agric..

[37] Greige-Gerges H., Khalil R.A., Mansour E.A., Magdalou J., Chahine R., Ouaini N. (2007). Cucurbitacins from *Ecballium elaterium* juice increase the binding of bilirubin and ibuprofen to albumin in human plasma. Chem. Biol. Interact..

[38] Seger C., Sturm S., Haslinger E., Stuppner H. (2005). NMR Signal Assignment of 22-Deoxocucurbitacin D and Cucurbitacin D from *Ecballium elaterium* L. (*Cucurbitaceae*). Monatsh. Chem..

[39] Hano Y., Shi Y.Q., Nomura T., Yang P.Q., Chang W.J. (1997). Two acetogenins from *Hemsleya ellipsoidea*. Phytochemistry.

[40] Torres-Moreno H., Marcotullio M.C., Velazquez C., Ianni F., Garibay-Escobar A., Robles-Zepeda R.E. (2020). Cucurbitacin IIb, a steroidal triterpene from *Ibervillea sonorae* induces antiproliferative and apoptotic effects on cervical and lung cancer cells. Steroids.

[41] Attar U.A., Ghane S.G. (2018). Optimized extraction of anti-cancer compound—Cucurbitacin I and LC–MS identification of major metabolites from wild Bottle gourd (*Lagenaria siceraria* (Molina) Standl.). S. Afr. J. Bot..

[42] Kumar S., Sharma K., Sahai M., Maurya R. (2019). A New Cucurbitacin Glucoside from *Luffa graveolense*. Chem. Nat. Compd..

[43] Liu J.Q., Chen J.C., Wang C.F., Qiu M.H. (2009). New Cucurbitane Triterpenoids and Steroidal Glycoside from *Momordica charantia*. Molecules.

[44] Aguiniga-Sanchez I., Cadena-Iniguez J., Santiago-Osorio E., Gomez-Garcia G., Mendoza-Nunez V.M., Rosado-Perez J., Ruiz-Ramos M., Cisneros-Solano V.M., Ledesma-Martinez E., Delgado-Bordonave A.D. (2017). Chemical analyses and in vitro and in vivo toxicity of fruit methanol extract of *Sechium edule* var. nigrum spinosum. Pharm. Biol..

[45] Kongtun S., Juratchariyakul W., Kummalue T., Tan-ariya P., Kunnachak S., Frahm A.W. (2009). Cytotoxic properties of root extract and fruit juice of *Trichosanthes cucumerina*. Planta Med..

[46] Ryu S.Y., Lee S.H., Choi S.U., Lee C.O., No Z., Ahn J.W. (1994). Antitumor activity of *Trichosanthes kirilowii*. Arch. Pharm. Res..

[47] Oh H., Mun Y.J., Im S.J., Lee S.Y., Song H.J., Lee H.S., Woo W.H. (2002). Cucurbitacins from *Trichosanthes kirilowii* as the inhibitory components on tyrosinase activity and melanin synthesis of B16/F10 melanoma cells. Planta Med..

[48] Kanchanapoom T., Kasai R., Yamasaki K. (2002). Cucurbitane, hexanorcucurbitane and octanorcucurbitane glycosides from fruits of *Trichosanthes tricuspidata*. Phytochemistry.

[49] Frei B., Heinrich M., Herrmann D., Orjala J.E., Schmitt J., Sticher O. (1998). Phytochemical and biological investigation of *Begonia heracleifolia*. Planta Med..

[50] Wu P.L., Lin F.W., Wu T.S., Kuoh C.H., Lee K.H., Lee S.J. (2004). Cytotoxic and anti-HIV principles from the rhizomes of *Begonia nantoensis*. Chem. Pharm. Bull..

[51] Sachdev-Gupta K., Radke C.D., Renwick J.A.A. (1993). Antifeedant activity of cucurbitacins from *Iberis amara* against larvae of *Pieris rapae*. Phytochemistry.

[52] Curtis P.J., Meade P.M. (1971). Cuburbitacins from the *Cruciferae*. Phytochemistry.

[53] Sarker S.D., Whiting P., Lafont R., Girault J.P., Dinan L. (1997). Cucurbitacin D from *Cercidiphyllum japonicum*. Biochem. Syst. Ecol..

[54] Sasamori H., Reddy K.S., Kirkup M.P., Shabanowitz J., Lynn D.G., Hecht S.M., Woode K.A., Bryan R.F., Campbell J., Lynn W.S. (1983). New cytotoxic principles from *Datisca glomerata*. J. Chem. Soc. Perkin Trans..

[55] Reddy K.S., Amonkar A.J., McCloud T.G., Chang C.J., Cassady J.M. (1988). Spinosides A and B. Two cytotoxic cucurbitacin glycosides from *Desfontainia spinosa*. Phytochemistry.

[56] Oberlies N.H., Burgess J.P., Navarro H.A., Pinos R.E., Soejarto D.D., Farnsworth N.R., Kinghorn A.D., Wani M.C., Wall M.E. (2001). Bioactive constituents of the roots of *Licania intrapetiolaris*. J. Nat. Prod..

[57] Meng D., Qiang S., Lou L., Zhao W. (2008). Cytotoxic cucurbitane-type triterpenoids from *Elaeocarpus hainanensis*. Planta Med..

[58] Ito A., Chai H.B., Lee D., Kardono L.B.S., Riswan S., Farnsworth N.R., Cordell G.A., Pezzuto J.M., Kinghorn A.D. (2002). Ellagic acid derivatives and cytotoxic cucurbitacins from *Elaeocarpus mastersii*. Phytochemistry.

[59] Wang Y.J., Yang J., Li X.N., Bai H., Luo J.F., He Z.R., Wang Y.H. (2022). Cucurbitane-type triterpenoids from the branches and leaves of *Elaeocarpus sylvestris*. Phytochem. Lett..

[60] Rodriguez N., Vasquez Y., Hussein A.A., Coley P.D., Solis P.N., Gupta M.P. (2003). Cytotoxic cucurbitacin constituents from *Sloanea zuliaensis*. J. Nat. Prod..

[61] Liu M.T., Lin S., Wang Y.H., He W.Y., Li S., Wang S.J., Yang Y.C., Shi J.G. (2007). Two novel glycosidic triterpene alkaloids from the stem barks of *Machilus yaoshansis*. Org. Lett..

[62] Gan M., Liu M., Liu B., Lin S., Zhang Y., Zi J., Song W., Ye F., Chen X., Shi J. (2011). Cucurbitane glucosides from the root of *Machilus yaoshansis*. J. Nat. Prod..

[63] Bean M.F., Antoun M., Abramson D., Chang C.J., McLaughlin J.L., Cassady J.M. (1985). Cucurbitacin B and Isocucurbitacin B: Cytotoxic Components of *Helicteres isora*. J. Nat. Prod..

[64] Bhandari P., Kumar N., Singh B., Kaul V.K. (2007). Cucurbitacins from *Bacopa monnieri*. Phytochemistry.

[65] Musza L.L., Speight P., McElhiney S., Barrow C.J., Gillum A.M., Cooper R., Killar L.M. (1994). Cucurbitacins, cell adhesion inhibitors from *Conobea scoparioides*. J. Nat. Prod..

[66] Kim I.H., Uchiyama N., Kawahara N., Goda Y. (2006). Iridoid glycosides and cucurbitacin glycoside from *Neopicrorhiza scrophulariiflora*. Phytochemistry.

[67] Huang Y., De Bruyne T., Apers S., Ma Y., Claeys M., Vanden Berghe D., Pieters L., Vlietinck A. (1998). Complement-inhibiting cucurbitacin glycosides from *Picria fel-terrae*. J. Nat. Prod..

[68] Stuppner H., Wagner H. (1989). New cucurbitacin glycosides from *Picrorhiza kurrooa*. Planta Med..

[69] Stuppner H., Moller E.P. (1993). Cucurbitacins with unusual side chains from *Picrorhiza kurroa*. Phytochemistry.

[70] Wang H., Ye W.C., Zhao S.X. (2004). Cucurbitacin glycosides and the monoterpene jiofuran from *Picrorhiza scrophulariiflora*. Biochem. Syst. Ecol..

[71] Arisawa M., Pezzuto J.M., Kinghorn A.D., Cordell G.A., Farnsworth N.R. (1984). Plant Anticancer Agents XXX: Cucurbitacins from *Ipomopsis aggregata* (*Polemoniaceae*). J. Pharm. Sci..

[72] Yamada Y., Hagiwara K., Iguchi K., Suzuki S., Hsu H.Y. (1978). Isolation and structures of arvenins from *Anagallis arvensis* L. (*Primulaceae*). New cucurbitacin glucosides. Chem. Pharm. Bull..

[73] Munoz O., Estevez-Braun A.M., Ravelo A.G., Gonzalez A.G. (2002). Cucurbitacin F in seeds of *Kageneckia angustifolia* (*Rosaceae*). Z. Naturforsch..

[74] Munoz O., Delporte C., Backhouse N., Erazo S., Negrete R., Maldonado S., Lopez-Perez J.L., San Feliciano A. (2000). A new cucurbitacin glycoside from *Kageneckia oblonga* (*Rosaceae*). Z. Naturforsch..

[75] Maloney K.N., Fujita M., Eggert U.S., Schroeder F.C., Field C.M., Mitchison T.J., Clardy J. (2008). Actin-Aggregating Cucurbitacins from *Physocarpus capitatus*. J. Nat. Prod..

[76] Sarker S.D., Whiting P., Sik V., Dinan L. (1999). Ecdysteroid antagonists (cucurbitacins) from *Physocarpus opulifolius* (*Rosaceae*). Phytochemistry.

[77] Konoshima T., Takasaki M., Kozuka M., Haruna M., Ito K., Estes J.R., Lee K.H. (1993). Constituents of rosaceous plants. I. Structure of new triterpenoids from *Cowania mexicana*. Chem. Pharm. Bull..

[78] Kim D.K., Choi S.H., Lee J.O., Ryu S.Y., Park D.K., Shin D.H., Jung J.H., Pyo S.K., Lee K.R., Zee O.P. (1997). Cytotoxic constituents of *Sorbaria sorbifolia* var. stellipila. Arch. Pharm. Res..

[79] Guerrero-Analco J.A., Hersch-Martinez P., Pedraza-Chaverri J., Navarrete A., Mata R. (2005). Antihyperglycemic effect of constituents from *Hintonia standleyana* in streptozotocin-induced diabetic rats. Planta Med..

[80] Mata R., Rios L., Rayo Camacho D., Reguero M.T., Lorence D. (1988). Triterpenes from *Cigarrilla mexicana*. Phytochemistry.

[81] Mata R., Castaneda P., Camacho M., Delgado G. (1988). Chemical studies on Mexican plants used in traditional medicine, V. Cucurbitacin glucosides from *Cigarrilla mexicana*. J. Nat. Prod..

[82] Sun J., Xia F., Wang S., Wang K.Y., Chen J.M., Tu P.F. (2015). Structural elucidation of two new megastigmane glycosides from the leaves of *Aquilaria sinensis*. Chin. J. Nat. Med..

[83] Fuller R.W., Cardellina J.H., Cragg G.M., Boyd M.R. (1994). Cucurbitacins: Differential cytotoxicity, dereplication and first isolation from *Gonystylus keithii*. J. Nat. Prod..

[84] Bashir S.S., Hussain A., Hussain S.J., Wani O.A., Nabi S.Z., Dar N.A., Baloch F.S., Mansoor S. (2021). Plant drought stress tolerance: Understanding its physiological, biochemical and molecular mechanisms. Biotechnol. Biotechnol. Equip..

[85] Aparna, Skarzyńska A., Pląder W., Pawełkowicz M. (2023). Impact of Climate Change on Regulation of Genes Involved in Sex Determination and Fruit Production in Cucumber. Plants.

[86] Shang Y., Ma Y., Zhou Y., Zhang H., Duan L., Chen H., Zeng J., Zhou Q., Wang S., Gu W. (2014). Biosynthesis, regulation, and domestication of bitterness in cucumber. Science.

[87] Kano Y., Goto H. (2003). Relationship between the occurrence of bitter fruit in cucumber (*Cucumis sativus* L.) and the contents of total nitrogen, amino acid nitrogen, protein and HMG-CoA reductase activity. Sci. Hortic..

[88] Zhao G., Wang M., Luo C., Li J., Gong H., Zheng X., Liu X., Luo J., Wu H. (2022). Metabolome and Transcriptome Analyses of Cucurbitacin Biosynthesis in Luffa (*Luffa acutangula*). Front. Plant. Sci..

[89] Mashilo J., Odindo A.O., Shimelis H.A., Musenge P., Tesfay S.Z., Magwaza L.S. (2018). Photosynthetic response of bottle gourd [*Lagenaria siceraria* (Molina) Standl.] to drought stress: Relationship between cucurbitacins accumulation and drought tolerance. Sci. Hortic..

[90] Wang W.D., Liu Y., Su Y., Xiong X.Z., Shang D., Xu J.J., Liu H.J. (2017). Antitumor and apoptotic effects of cucurbitacin a in A-549 lung carcinoma cells is mediated via G2/M cell cycle arrest and M-TOR/PI3K/Akt signalling pathway. Afr. J. Tradit. Complement. Altern. Med..

[91] Liu J.Y., Liu X., Ma W., Kou W., Li C.L., Zhao J. (2018). Anticancer activity of cucurbitacin-A in ovarian cancer cell line SKOV3 involves cell cycle arrest, apoptosis and inhibition of mTOR/PI3K/Akt signaling pathway. J. BUON..

[92] Luo W.W., Zhao W.W., Lu J.J., Wang Y.T., Chen X.P. (2018). Cucurbitacin B suppresses metastasis mediated by reactive oxygen species (ROS) via focal adhesion kinase (FAK) in breast cancer MDA-MB-231 cells. Chin. J. Nat. Med..

[93] Guo J., Wu G., Bao J., Hao W., Lu J., Chen X. (2014). Cucurbitacin B induced ATM-mediated DNA damage causes G2/M cell cycle arrest in a ROS-dependent manner. PLoS ONE.

[94] Aribi A., Gery S., Lee D.H., Thoennissen N.H., Thoennissen G.B., Alvarez R., Ho Q., Doan N.B., Chan K.T., Toh M. (2013). The triterpenoid cucurbitacin B augments the antiproliferative activity of chemotherapy in human breast cancer. Int. J. Cancer.

[95] Zhang M., Yin L., Yang S., Hong J., Chen C., Han D., Hou Y., Zhang B., Huang L., Zhang A. (2012). Abstract 5728: The synergistic effect of Cucurbitacin B and radiation treatment. Cancer Res..

[96] Bakar F. (2016). Cucurbitacin B Enhances the Anticancer Effect of Imatinib Mesylate Through Inhibition of MMP-2 Expression in MCF-7 and SW480 Tumor Cell Lines. Anticancer. Agents Med. Chem..

[97] Kaewmeesri P., Pocasap P., Kukongviriyapan V., Prawan A., Kongpetch S., Senggunprai L. (2022). Anti-metastatic Potential of Natural Triterpenoid Cucurbitacin B Against Cholangiocarcinoma Cells by Targeting Src Protein. Integr. Cancer Ther..

[98] Yar Saglam A.S., Alp E., Elmazoglu Z., Menevse S. (2016). Treatment with cucurbitacin B alone and in combination with gefitinib induces cell cycle inhibition and apoptosis via EGFR and JAK/STAT pathway in human colorectal cancer cell lines. Hum. Exp. Toxicol..

[99] Liu X., Duan C., Ji J., Zhang T., Yuan X., Zhang Y., Ma W., Yang J., Yang L., Jiang Z. (2017). Cucurbitacin B induces autophagy and apoptosis by suppressing CIP2A/PP2A/mTORC1 signaling axis in human cisplatin resistant gastric cancer cells. Oncol. Rep..

[100] Yin D., Wakimoto N., Xing H.T., Lu D., Huynh T., Wang X., Black K.L., Koeffler H.P. (2008). Cucurbitacin B markedly inhibits growth and rapidly affects the cytoskeleton in glioblastoma multiforme. Int. J. Cancer.

[101] Sun Y., Zhang J., Zhou J., Huang Z., Hu H., Qiao M., Zhao X., Chen D. (2015). Synergistic effect of cucurbitacin B in combination with curcumin via enhancing apoptosis induction and reversing multidrug resistance in human hepatoma cells. Eur. J. Pharmacol..

[102] Touihri-Barakati I., Kallech-Ziri O., Ayadi W., Kovacic H., Hanchi B., Hosni K., Luis J. (2017). Cucurbitacin B purifed from *Ecballium elaterium* (L.) A. Rich from Tunisia inhibits alpha5beta1 integrin-mediated adhesion, migration, proliferation of human glioblastoma cell line and angiogenesis. Eur. J. Pharmacol..

[103] Liu T., Zhang M., Zhang H., Sun C., Yang X., Deng Y., Ji W. (2008). Combined antitumor activity of cucurbitacin B and docetaxel in laryngeal cancer. Eur. J. Pharmacol..

[104] Liu J.H., Li C., Cao L., Zhang C.H., Zhang Z.H. (2022). Cucurbitacin B regulates lung cancer cell proliferation and apoptosis via inhibiting the IL-6/STAT3 pathway through the lncRNA XIST/miRlet-7c axis. Pharmaceutic. Biol..

[105] Yuan R., Fan Q., Liang X., Han S., He J., Wang Q.Q., Gao H., Feng Y., Yang S. (2022). Cucurbitacin B inhibits TGF-β1-induced epithelial-mesenchymal transition (EMT) in NSCLC through regulating ROS and PI3K/Akt/mTOR pathways. Chin. Med..

[106] Yu B., Zheng L., Tang H., Wang W., Lin Y. (2021). Cucurbitacin B enhances apoptosis in geftinib resistant non-small cell lung cancer by modulating the miR-17-5p/STAT3 axis. Mol. Med. Rep..

[107] Ding X., Chi J., Yang X., Hao J., Liu C., Zhu C., Wang X., Liu X., Niu Y., Ji W. (2017). Cucurbitacin B synergistically enhances the apoptosis-inducing effect of arsenic trioxide by inhibiting STAT3 phosphorylation in lymphoma Ramos cells. Leuk. Lymphoma.

[108] Wei J., Chen X., Li Y., Li R., Bao K., Liao L., Xie Y., Yang T., Zhu J., Mao F. (2022). Cucurbitacin B-induced G2/M cell cycle arrest of conjunctival melanoma cells mediated by GRP78-FOXM1-KIF20A pathway. Acta Pharmaceut. Sin. B.

[109] Zheng Q., Liu Y., Liu W., Ma F., Zhou Y., Chen M., Chang J., Wang Y., Yang G., He G. (2014). Cucurbitacin B inhibits growth and induces apoptosis through the JAK2/STAT3 and MAPK pathways in SHSY5Y human neuroblastoma cells. Mol. Med. Rep..

[110] Zhang Z.R., Gao M.X., Yang K. (2017). Cucurbitacin B inhibits cell proliferation and induces apoptosis in human osteosarcoma cells via modulation of the JAK2/STAT3 and MAPK pathways. Expert. Therap. Med..

[111] Lee D.H., Thoennissen N.H., Goff C., Iwanski G.B., Forscher C., Doan N.B., Said J.W., Koeffler H.P. (2011). Synergistic effect of low-dose cucurbitacin B and low-dose methotrexate for treatment of human osteosarcoma. Cancer Lett..

[112] El-Senduny F.F., Badria F.A., El-Waseef A.M., Chauhan S.C., Halaweish F. (2016). Approach for chemosensitization of cisplatin-resistant ovarian cancer by cucurbitacin B. Tumor Biol..

[113] Zhou J., Zhao T., Ma L., Liang M., Guo Y.J., Zhao L.M. (2017). Cucurbitacin B and SCH772984 exhibit synergistic anti-pancreatic cancer activities by suppressing EGFR, PI3K/Akt/mTOR, STAT3 and ERK signaling. Oncotarget.

[114] Thoennissen N.H., Iwanski G.B., Doan N.B., Okamoto R., Lin P., Abbassi S., Song J.H., Yin D., Toh M., Xie W.D. (2009). Cucurbitacin B Induces Apoptosis by Inhibition of the *JAK/STAT* Pathway and Potentiates Antiproliferative Effects of Gemcitabine on Pancreatic Cancer Cells. Cancer Res..

[115] Iwanski G.B., Lee D.H., En-Gal S., Doan N.B., Castor B., Vogt M., Toh M., Bokemeyer C., Said J.W., Thoennissen N.H. (2010). Cucurbitacin B, a novel in vivo potentiator of gemcitabine with low toxicity in the treatment of pancreatic cancer. Br. J. Pharmacol..

[116] Tao B., Wang D., Yang S., Liu Y., Wu H., Li Z., Chang L., Yang Z., Liu W. (2021). Cucurbitacin B Inhibits Cell Proliferation by Regulating X-Inactive Specific Transcript Expression in Tongue Cancer. Front. Oncol..

[117] Ku J.M., Kim S.R., Hong S.H., Choi H.S., Seo H.S., Shin Y.C., Ko S.G. (2015). Cucurbitacin D induces cell cycle arrest and apoptosis by inhibiting STAT3 and NF-κB signaling in doxorubicin-resistant human breast carcinoma (MCF7/ADR) cells. Mol. Cell Biochem..

[118] Sikander M., Hafeez B.B., Malik S., Alsayari A., Halaweish F.T., Yallapu M.M., Chauhan S.C., Jaggi M. (2016). Cucurbitacin D exhibits potent anti-cancer activity in cervical cancer. Sci. Rep..

[119] Sikander M., Malik S., Khan S., Kumari S., Chauhan N., Khan P., Halaweish F.T., Chauhan B., Yallapu M.M., Jaggi M. (2019). Novel Mechanistic Insight into the Anticancer Activity of Cucurbitacin D against Pancreatic Cancer (Cuc D Attenuates Pancreatic Cancer). Cells.

[120] Sikander M., Malik S., Hafeez B.B., Mandil H., Halaweish F.T., Jaggi M., Chauhan S.C. (2018). Abstract 2934: Cucurbitacin D enhances the therapeutic efficacy of docetaxel via targeting cancer stem cells and miR-145. Cancer Res..

[121] Jacquot C., Rousseau B., Carbonnelle D., Chinou I., Malleter M., Tomasoni C., Roussakis C. (2014). Cucurbitacin-D-induced CDK1 mRNA up-regulation causes proliferation arrest of a non-small cell lung carcinoma cell line (NSCLC-N6). Anticancer. Res..

[122] Cheng A.C., Hsu Y.C., Tsai C.C. (2019). The efects of cucurbitacin E on GADD45βtrigger G2/M arrest and JNK-independent pathway in brain cancer cells. J. Cell Mol. Med..

[123] Jing S.Y., Wu Z.D., Zhang T.H., Zhang J., Wei Z.Y. (2020). In vitro antitumor efect of cucurbitacin E on human lung cancer cell line and its molecular mechanism. Chin. J. Nat. Med..

[124] Hsu Y.C., Chen M.J., Huang T.Y. (2014). Inducement of mitosis delay by cucurbitacin E, a novel tetracyclic triterpene from climbing stem of *Cucumis melo* L., through GADD45gamma in human brain malignant glioma (GBM) 8401 cells. Cell Death Dis..

[125] Lan T., Wang L.L., Xu Q., Liu W.G., Jin H.C., Mao W.M., Wang X., Wang X. (2013). Growth inhibitory effect of cucurbitacin E on breast cancer cells. Int. J. Clin. Exp. Pathol..

[126] Si W., Lyu J., Liu Z., Wang C., Huang J., Jiang L., Ma T. (2019). Cucurbitacin E inhibits cellular proliferation and enhances the chemo-response in gastric cancer by suppressing AKt activation. J. Cancer.

[127] Eyol E., Tanriverdi Z., Karakus F., Yilmaz K., Unuvar S. (2016). Synergistic Anti-proliferative Effects of Cucurbitacin I and Irinotecan on Human Colorectal Cancer Cell Lines. Clin. Exp. Pharmacol..

[128] Song J.M., Liu H.X., Li Z., Yang C., Wang C.J. (2015). Cucurbitacin I inhibits cell migration and invasion and enhances chemosensitivity in colon cancer. Oncol. Rep..

[129] Kim H.J., Park J.H.P., Kim J.K. (2014). Cucurbitacin-I, a natural cell-permeable triterpenoid isolated from *Cucurbitaceae*, exerts potent anticancer effect in colon cancer. Chem. Biol. Interact..

[130] Zhu X., Huang H., Zhang J., Liu H., Ao R., Xiao M., Wu Y. (2018). The anticancer effects of Cucurbitacin I inhibited cell growth of human non small cell lung cancer through PI3K/AKT/p70S6K pathway. Mol. Med. Rep..

[131] Ni Y., Wu S., Wang X., Zhu G., Chen X., Ding Y., Jiang W. (2018). Cucurbitacin I induces pro-death autophagy in A549 cells via the ERK-mTOR-STAT3 signaling pathway. J. Cell. Biochem..

[132] Li H., Chen H., Li R., Xin J., Wu S., Lan J., Xue K., Li X., Zuo C., Jiang W. (2019). Cucurbitacin I induces cancer cell death through the endoplasmic reticulum stress pathway. J. Cell. Biochem..

[133] Sadzuka Y., Fujiki S., Itai S. (2012). Enhancement of doxorubicin-induced antitumor activity and reduction of adverse reactions by cucurbitacin I. Food Res. Int..

[134] Xu D., Shen H., Tian M., Chen W., Zhang X. (2022). Cucurbitacin I inhibits the proliferation of pancreatic cancer through the JAK2/STAT3 signalling pathway in vivo and in vitro. J. Cancer.

[135] Zhang J., Song Y., Liang Y., Zou H., Zuo P., Yan M., Jing S., Li T., Wang Y., Li D. (2019). Cucurbitacin IIa interferes with EGFR-MAPK signaling pathway leads to proliferation inhibition in A549cells. Food. Chem. Toxicol..

[136] Liang Y., Zhang T., Ren L., Jing S., Li Z., Zuo P., Li T., Wang Y., Zhang J., Wei Z. (2021). Cucurbitacin IIb induces apoptosis and cell cycle arrest through regulating EGFR/MAPK pathway. Environ. Toxicol. Pharmacol..

[137] Arjaibi H.M., Ahmed M.S., Halaweish F.T. (2017). Mechanistic investigation of hepato-protective potential for cucurbitacins. Med. Chem. Res..

[138] Zhong H., Huang Y., Deng X., Liu M., Luo W. (2020). Cucurbitacin B supplementation reduces inflammatory responses and alveolar bone loss via regulating MPO, COX-2 and RANK/RANKL/OPG signals in a rodent model of ligature-induced periodontitis. J. King Saud. Univ.—Sci..

[139] Shawkey A.M., Rabeh M.A., Abdellatif A.O. (2014). Biofunctional molecules from *Citrullus colocynthis*: An HPLC/MS analysis in correlation to antimicrobial and anticancer activities. Advan. Life Sci. Tech..

[140] Kapoor N., Ghorai S.M., Kushwaha P.K., Shukla R., Aggarwal C., Bandichhor R. (2020). Plausible mechanisms explaining the role of cucurbitacins as potential therapeutic drugs against coronavirus 2019. Inform. Med. Unlocked.

[141] Xiao Y., Yang Z., Wu Q.Q., Jiang X.H., Yuan Y., Chang W., Bian Z.Y., Zhu J.X., Tang Q.Z. (2017). Cucurbitacin B Protects Against Pressure Overload Induced Cardiac Hypertrophy. J. Cell. Biochem..

[142] Hernández Navia S.E., Figueroa-Hernández J.L., Rodriguez-Zavala J.S., Rodriguez-Sosa M., Martinez-Vasquez M. (2022). Anti-Diabetic Effects of Cucurbitacins from *Ibervillea lindheimeri* on Induced Mouse Diabetes. J. Chem..

[143] Kumar A., Sharma B., Sharma U., Parashar G., Parashar N.C., Rani I., Ramniwas S., Kaur S., Haque S., Tuli H.S. (2023). Apoptotic and antimetastatic effect of cucurbitacins in cancer: Recent trends and advancement. Naunyn Schmiedebergs Arch. Pharmacol..

[144] Yung M.M.H., Ross F.A., Hardie D.G., Leung T.H., Zhan J., Ngan H.Y.S., Chan D.W. (2016). Bitter melon (*Momordica charantia*) extract functions as a natural AMPK activator and synergistically enhances cisplatin cytotoxicity in ovarian cancer cells. Integr. Cancer Ther..

[145] Gou M., Wei X., Men K., Wang B., Luo F., Zhao X., Wei Y.Q., Qian Z.Y. (2011). PCL/PEG copolymeric nanoparticles: Potential nanoplatforms for anticancer agent delivery. Curr. Drug Targets..

[146] Chountoulesi M., Selianitis D., Pispas S., Pippa N. (2023). Recent Advances on PEO-PCL Block and Graft Copolymers as Nanocarriers for Drug Delivery Applications. Materials.

[147] Boykin C., Zhang G., Chen Y.H., Zhang R.W., Fan X.E., Yang W.M., Lu Q. (2011). Cucurbitacin IIa: A novel class of anti-cancer drug inducing non-reversible actin aggregation and inhibiting survivin independent of JAK2/STAT3 phosphorylation. Br. J. Cancer.

[148] Cai Y., Fang X., He C., Li P., Xiao F., Wang Y., Chen M. (2015). Cucurbitacins: A systematic review of the phytochemistry and anticancer activity. Am. J. Chin. Med..

[149] Kim S.R., Seo H.S., Choi H.S., Cho S.G., Kim Y.K., Hong E.H., Shin Y.C., Ko S.G. (2013). *Trichosanthes kirilowii* ethanol extract and cucurbitacin D inhibit cell growth and induce apoptosis through inhibition of STAT3 activity in breast cancer cells. Evid. Based Complement. Alternat. Med..

[150] Sun J., Blaskovich M.A., Jove R., Livingstone S.K., Coppola D., Sebti S.M. (2005). Cucurbitacin Q: A selective STAT3 activation inhibitor with potent antitumor activity. Oncogene.

[151] Üremiş M.M., Üremiş N., Türköz Y. (2023). Cucurbitacin E shows synergistic effect with sorafenib by inducing apoptosis in hepatocellular carcinoma cells and regulates Jak/Stat3, ERK/MAPK, PI3K/Akt/mTOR signaling pathways. Steroids.

[152] Duangmano S., Sae-Lim P., Suksamrarn A., Patmasiriwat P., Domann F.E. (2012). Cucurbitacin B causes increased radiation sensitivity of human breast cancer cells via G2/M cell cycle arrest. J. Oncol..

[153] Kim M.S., Lee K., Ku J.M., Choi Y.J., Mok K., Kim D., Cheon C., Ko S.G. (2020). Cucurbitacin D induces G2/M phase arrest and apoptosis via the ROS/p38 pathway in Capan-1 pancreatic cancer cell line. Evid. Based Complement. Alternat. Med..

[154] Xie Y.L., Tao W.H., Yang T.X., Qiao J.G. (2016). Anticancer effect of cucurbitacin B on MKN-45 cells via inhibition of the JAK2/STAT3 signaling pathway. Exp. Ther. Med..

[155] Ren G., Sha T., Guo J., Li W., Lu J., Chen X. (2015). Cucurbitacin B induces DNA damage and autophagy mediated by reactive oxygen species (ROS) in MCF-7 breast cancer cells. J. Nat. Med..

[156] Silva V.R., Santos L.D.S., Dias R.B., Quadros C.A., Bezerra D.P. (2021). Emerging agents that target signaling pathways to eradicate colorectal cancer stem cells. Cancer Commun..

[157] Silvestre G.F.G., de Lucena R.P., da Silva Alves H. (2022). Cucurbitacins and the immune system: Update in research on anti-inflammatory, antioxidant, and immunomodulatory mechanisms. Curr. Med. Chem..

[158] Rymal K.S., Chambliss O.L., Bond M.D., Smith D.A. (1984). Squash Containing Toxic Cucurbitacin Compounds Occurring in California and Alabama. J. Food. Prot..

[159] David A., Vallance D.K. (1955). Bitter Principles of *Cucurbitaceae*. J. Pharm. Pharmacol..

[160] Jorn G., Inge S., Hans C.A. (2006). Cucurbitacins in Plant Food.

[161] Tamura Y., Maki T., Kan K., Nagayama T., Naoi Y. (1983). Outbreaks of food poisoning through chemicals and natural toxicants in Tokyo. I. 1980–1982. Ann. Rep. Tokyo Metro. Res. Lab. Public Health.

[162] Edery H., Schatzberg-Porath G., Gitter S. (1961). Pharmacodynamic activity of elatericin (Cucurbitacin D). Arch. Int. Pharmacodyn. Ther..

[163] Njoroge G.N., Leonard E.N. (1994). Edible and poisionous species of cucurbitaceae in the central highlands of Kenya. J. East. Afr. Nat. Hist..

[164] Yaowalak U., Usaneeporn L., Weena J., Tanawan K. (2010). Immunosuppressive effects of Cucurbitacin B on human peripheral blood lymphocytes. J. Med. Plants Res..

[165] Dai S., Wang C., Zhao X., Ma C., Fu K., Liu Y., Peng C., Li Y. (2023). Cucurbitacin B: A Review of Its Pharmacology, Toxicity, and Pharmacokinetics. Pharmacol. Res..

[166] Wang Z., Zhu W., Gao M., Wu C., Yang C., Yang J., Wu G., Yang B., Kuang H. (2017). Simultaneous Determination of Cucurbitacin B and Cucurbitacin E in Rat Plasma by UHPLC-MS/MS: A Pharmacokinetics Study after Oral Administration of Cucurbitacin Tablets. J. Chromatogr. B Analyt. Technol. Biomed. Life Sci..

[167] Xiao Y., Zhao Q., Wu Q., Chang J., Xue H., Liu C., Liu X. (2018). A New Sensitive UPLC-MS/MS Method for the Determination of Cucurbitacin B in Rat Plasma: Application to an Absolute Bioavailability Study. RSC Adv..

[168] Hunsakunachai N., Nuengchamnong N., Jiratchariyakul W., Kummalue T., Khemawoot P. (2019). Pharmacokinetics of Cucurbitacin B from *Trichosanthes cucumerina* L. in Rats. BMC Complement. Altern. Med..

[169] Tang L., Fu L., Zhu Z., Yang Y., Sun B., Shan W., Zhang Z. (2018). Modified Mixed Nanomicelles with Collagen Peptides Enhanced Oral Absorption of Cucurbitacin B: Preparation and Evaluation. Drug Deliv..

[170] Garg S., Kaul S.C., Wadhwa R. (2018). Cucurbitacin B and cancer intervention: Chemistry, biology and mechanisms (Review). Int. J. Oncol..

[171] Marostica L.L., de Barros A.L.B., Oliveira J., Salgado B.S., Cassali G.D., Leite E.A., Cardoso V.N., Lang K.L., Caro M.S.B., Duran F.J. (2017). Antitumor effectiveness of a combined therapy with a new cucurbitacin B derivative and paclitaxel on a human lung cancer xenograft model. Toxicol. Appl. Pharmacol..

[172] Liu T., Peng H., Zhang M., Deng Y., Wu Z. (2010). Cucurbitacin B, a small molecule inhibitor of the Stat3 signaling pathway, enhances the chemosensitivity of laryngeal squamous cell carcinoma cells to cisplatin. Eur. J. Pharmacol..

[173] Kim S.H., Kang J.G., Kim C.S., Ihm S.H., Choi M.G., Yoo H.J., Lee S.J. (2017). Doxorubicin has a synergistic cytotoxicity with cucurbitacin B in anaplastic thyroid carcinoma cells. Tumour Biol..

